# Allele-specific CRISPR-Cas9 editing of dominant epidermolysis bullosa simplex in human epidermal stem cells

**DOI:** 10.1016/j.ymthe.2023.11.027

**Published:** 2023-12-05

**Authors:** C. Cattaneo, E. Enzo, L. De Rosa, L. Sercia, F. Consiglio, M. Forcato, S. Bicciato, A. Paiardini, G. Basso, E. Tagliafico, A. Paganelli, C. Fiorentini, C. Magnoni, M.C. Latella, M. De Luca

**Affiliations:** 1 Centre for Regenerative Medicine "Stefano Ferrari", Department of Life Sciences, University of Modena and Reggio Emilia, 41125 Modena, Italy; 2 Holostem Terapie Avanzate, s.r.l, 41125 Modena, Italy; 3 Department of Life Sciences, University of Modena and Reggio Emilia, 41125 Modena, Italy; 4 Department of Biochemical Sciences 'A. Rossi Fanelli', Sapienza Università di Roma, 00185 Rome, Italy; 5 Genomic Units, IRCCS Humanitas Research Hospital, 20089 Rozzano, Milan, Italy; 6 Department of Medical and Surgical Sciences, University of Modena and Reggio Emilia, 41124 Modena, Italy; 7 Regenerative and Oncological Dermatological Surgery Unit, Modena University Hospital, 41124 Modena, Italy

**Keywords:** epidermolysis bullosa, genetic disease, gene therapy, stem cells, keratinocytes biology, gene editing

## Abstract

Epidermolysis bullosa simplex (EBS) is a rare skin disease inherited mostly in an autosomal dominant manner. Patients display a skin fragility that leads to blisters and erosions caused by minor mechanical trauma. EBS phenotypic and genotypic variants are caused by genetic defects in intracellular proteins whose function is to provide the attachment of basal keratinocytes to the basement membrane zone and most EBS cases display mutations in keratin 5 (*KRT5*) and keratin 14 (*KRT14*) genes. Besides palliative treatments, there is still no long-lasting effective cure to correct the mutant gene and abolish the dominant negative effect of the pathogenic protein over its wild-type counterpart. Here, we propose a molecular strategy for EBS01 patient’s keratinocytes carrying a monoallelic c.475/495del21 mutation in *KRT14* exon 1. Through the CRISPR-Cas9 system, we perform a specific cleavage only on the mutant allele and restore a normal cellular phenotype and a correct intermediate filament network, without affecting the epidermal stem cell, referred to as holoclones, which play a crucial role in epidermal regeneration.

## Graphical abstract

**Figure:**
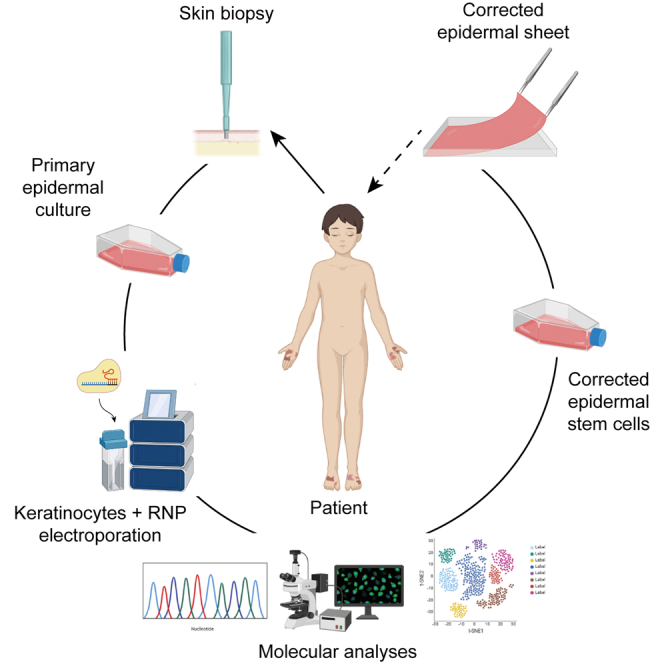

## Teaser

De Luca and colleagues propose an allele-specific gene-editing strategy for EBS01 patient affected by a rare skin disease inherited in an autosomal manner (EBS). This work aims to restore a normal cellular phenotype and a correct intermediate filament network in epidermal stem cells.

## Introduction

Inherited epidermolysis bullosa (EB) is a heterogeneous group of rare, autosomal genetic disorders caused by molecular defects within genes encoding structural proteins forming the epidermal-dermal junction. EB is characterized by recurrent blistering and erosions of the skin (and other stratified epithelia) that arise, spontaneously or upon minimal mechanical stress, within the epidermis in EB simplex (EBS), the lamina lucida in junctional EB (JEB) and beneath the lamina densa in dystrophic EB (DEB). EBS is the most common EB form, with a prevalence of 1 in 30,000 to 1 in 50,000.^1^^,^^2^ Its clinical manifestations are usually less severe than those of JEB and DEB, which can be devastating and even early lethal. However, some EBS forms are marked by a severe phenotype and several clinical variants have been identified based on the mutated gene, site of blister formation, anatomical distribution, and mode of inheritance.^3^^,^^4^^,^^5^

JEB and DEB are mostly recessively inherited, while the vast majority of EBS are inherited in a dominant manner. In fact, approximately 75% patients suffering from EBS harbors dominant mutations in *KRT5* and *KRT14*, the genes encoding keratin 5 (K5) and keratin 14 (K14), respectively. K5/K14 pairs form the basal keratinocyte intermediate filaments, which are part of the hemidesmosomal protein complex tethering the epidermal basal layer to the basement membrane and the underlying dermis. Mutant keratins exert a dominant negative effect on the functional keratins encoded by the normal allele, hence perturbing the basal keratinocyte intermediate filament network and leading to intraepidermal blister formation. Thus, while JEB and DEB can be tackled by the addition of a corrected copy of the mutated gene in the genome of epidermal stem cells,^6^^,^^7^^,^^8^^,^^9^^,^^10^^,^^11^^,^^12^^,^^13^^,^^14^ a potentially successful combined *ex vivo* cell and gene therapy of EBS strictly requires editing of the mutated allele.

Here, we outlined an allele-specific CRISPR-Cas9-based gene-editing approach that is able to disrupt specifically the *KRT14* mutant gene and fully restore functional intermediate filaments in epidermal stem cells cultivated from an EBS patient carrying a *de novo* monoallelic c.475/495del21 dominant mutation in exon 1 of *KRT14*.

This approach takes advantage of a tailored CRISPR-Cas9 system to induce double-strand breaks (DSBs) specifically on the mutant allele, leading to non-homologous end-joining (NHEJ) repairing process. These rearrangements are likely to generate frameshift mutations resulting in both pathogenic allele expression abolishment and phenotypic and mechanical stress resilience restoration. Besides the remarkable efficacy of this approach, we also demonstrate the correction of the epithelial stem cell compartment, which is mandatory for long-term skin regeneration. This highly effective and safe gene-editing strategy would therefore enable a translation to clinical application for the treatment of other dominant forms of EB.

## Results

### Novel monoallelic *KRT14* deletion causing a dominant form of EBS

**Figure 1:**
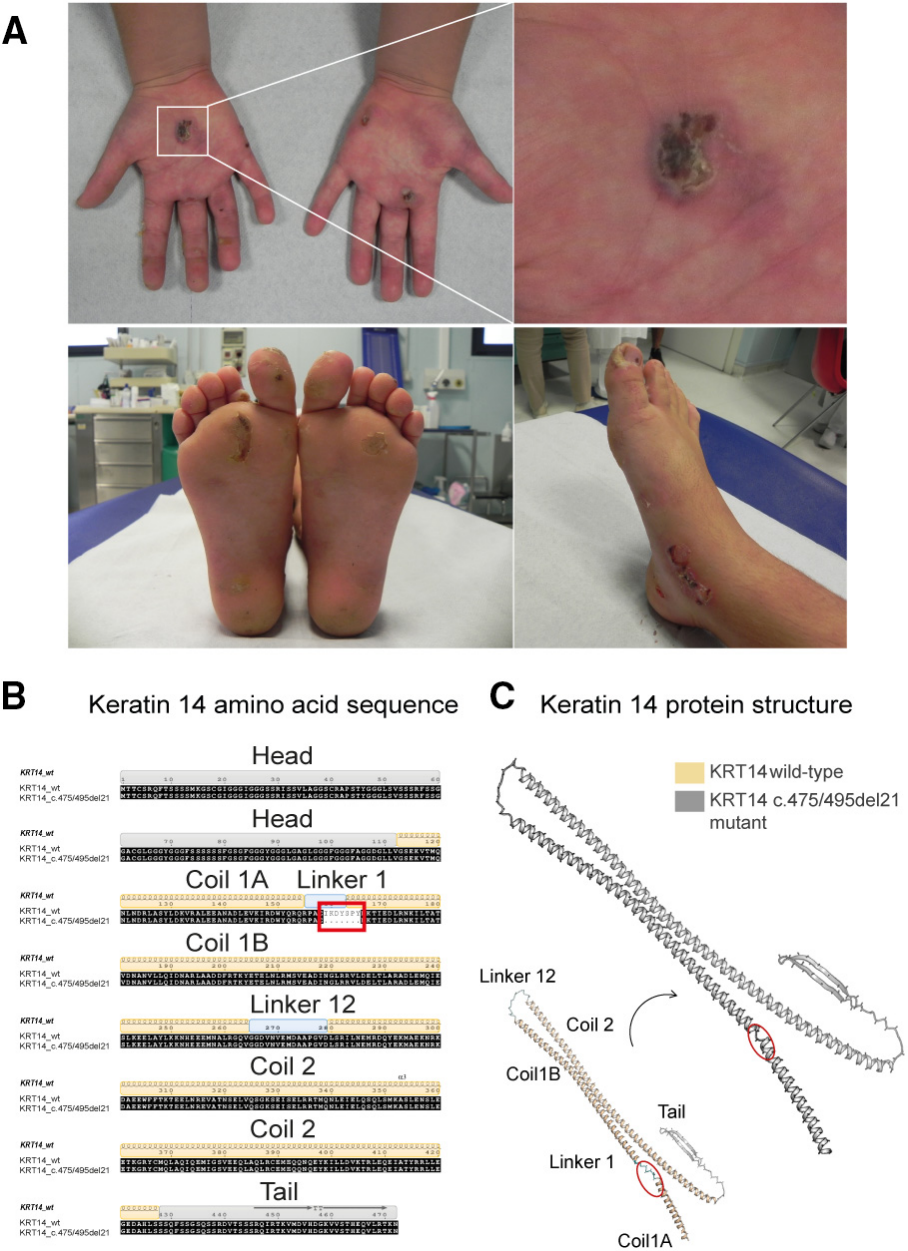
A novel monoallelic *KRT14* deletion causing a dominant form of EBS (A) Clinical images of the patient EBS01: bullous lesions are present on the palmoplantar region. Unremitting blister on the heel, dorsal medial part of the left foot, and right peri-malleolar region are shown in the panel below. (B) Amino acid sequence of the wild-type and mutant K14. The invariant residues are displayed in black boxes and the red box highlights the mutant region with the seven-amino-acid deletion. The secondary structures are represented above the sequences as ribbons (helices) and arrows (strands), respectively; blanks indicate unassigned regions. The head and tail domains of *KRT14* are represented as N-terminal and C-terminal gray boxes, respectively. Coil and linker regions are represented as yellow and cyan boxes, respectively. (C) Predicted three-dimensional structures of human wild-type (yellow) and mutant (gray) K14 protein. The lack of the linker 1 region, which is visible in the c475/495del21 protein, is circled in red.

An 8-year-old EBS patient (referred to as EBS01) suffered from a *de novo* heterozygous dominant mutation (c.475/495del21) within exon 1 of *KRT14*. The patient developed bullous skin lesions few months after birth and currently presents blisters in the palmoplantar region causing postural and ambulation problems. No other cases of EB were known among his relatives. Besides the palliative care and a regular multidisciplinary follow-up, no resolutive treatment is available for this patient (Figure 1A).

The EBS01 variant results in the deletion of seven in-frame amino acids, leading to a shorter K14 protein. The AlphaFold2^15^ suite for protein structure prediction and modeling was used to predict the 3D structure of the shorter K14 (Figure 1B). The *c.475/495del21* variant affects the protein structure resulting in the absence of an extended loop, linker L1, encompassing residues 159–165 (Figure 1B).^16^ The L1 structural motif of K14, whose function is still poorly characterized, is predicted to assume a highly flexible, non-helical β turn, with the pivotal function of connecting coil 1A with 1B (Figure 1C).^17^ The importance of the L1 motif is also reflected by its high evolutionary conservation in keratins.^18^ Indeed, mutations affecting the linker regions of intermediate filaments have previously been observed and related to severe cases of inherited skin-blistering diseases, highlighting the unexpected sensitivity of these regions to structural alterations.^17^

### Efficient and precise *KRT14* allele-specific editing in EBS primary keratinocytes

**Figure 2:**
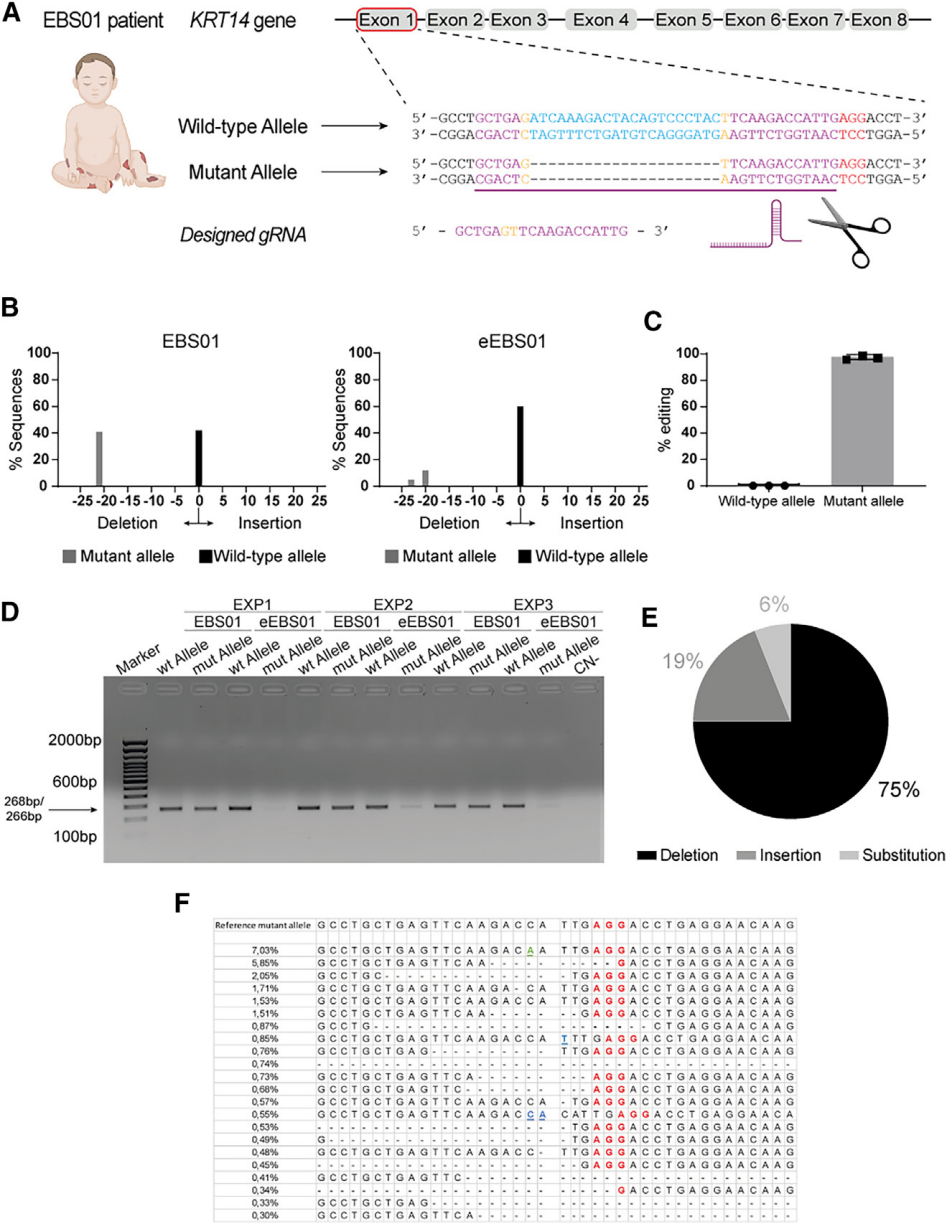
Gene editing of *KRT14* mutated allele (A) Representation of the EBS01 patient’s genotype and allele profile of *KRT14* gene on human chromosome 17. The expanded section of the exon 1 illustrates the 21 nucleotides absent in *KRT14* exon 1 mutant allele (light blue). Mutant allele-specific guide RNA is depicted in purple, straddling both the terminal sides of the deletion, and the AGG PAM sequence in red. (B) Graphic representation of TIDE analysis. Both the wild-type and mutant allele were detected in the untreated sample (EBS01). In the treated sample (eEBS01), the mutant allele has been deleted by gene editing. (C) Histogram representation of NGS analysis of three technical replicates post gene editing. Edited and not-edited percentages on both wild-type and mutant alleles are calculated on the total number of reads. (D) PCR analysis of three technical replicates (EXP1, EXP2, EXP3). For each experiment, a PCR product was amplified both from the EBS01 and eEBS01 keratinocytes’ genome with primers specific to anneal to wild-type (268 bp) and mutant alleles (266 bp). (E) Graphic representation of editing-derived indels in mutant *KRT14* allele (N = 3). (F) NGS analysis of PCR products surrounding the Cas9 target sites in the genome of eEBS01 showed a wide variety of indel mutations mediated by NHEJ at the targeted exon 1. The top sequence is the mutant allele unmodified sequence, and the dotted line represents the Cas9 cleavage upstream of the PAM.

The EBS01 genetic variation enables the mutant K14 to exert a dominant negative effect over its wild-type counterpart expressed by the other allele. Thus, a tailored CRISPR-Cas9 system was employed to target the monoallelic *de novo* mutation (*c.475/495del21*) within exon 1 of *KRT14* to induce a deleterious double-strand break on the mutant allele and promote NHEJ, inducing specific disruption of the mutant *KRT14* open reading frame. We designed a single guide RNA (sgRNA) tailored to specifically target only the mutant *KRT14* allele and employed the SpCas9 to specifically recognize the “NGG” PAM present near the deletion site. For the desired specificity, the 19-nucleotide-long guide RNA was sketched straddling both the terminal sides of the 21-base pair (bp) deletion and directly flanking the AGG PAM sequence within its 3′ end (Figure 2A).

To first assess the ability of the designed CRISPR-Cas9 system to specifically abolish the expression of the *KRT14* mutant allele, preliminary experiments employed a lentiviral vector to deliver the gene-editing machinery to EBS01-derived primary keratinocytes.

Strikingly, the gene-editing machinery was able to disrupt the expression of the mutant *KRT14* allele with an efficiency up to 94%, without affecting the wild-type allele (Figure S1A). We then performed an *in silico* genome analysis to assess the site specificity of sgRNA_del21 using Cas-OFFinder, followed by CCTop and COSMID. We could not detect off-target sites with either none or single mismatch and DNA bulge size = 0. However, introducing two random mismatches led to the discovery of two potential off targets, both located in intergenic sequences. When three random mismatches were introduced, we identified a total of 86 potential off targets. The majority of these off-target sites were found in intergenic regions (41) and introns (44), with only one potential off target located in a coding region (3′ untranslated region of *ZNF641* gene). We focused on six off targets out of these candidates, based on their potential relevance to gene function in the epidermis.

TIDE (Tracking of Indels by Decomposition) analysis outlined the presence of unwanted cleavage in one of the predicted off-target sites (Figure S1B). These preliminary data suggested that the editing strategy designed to tackle the EBS01 mutation is indeed appropriate.

Lentiviral-mediated genome integration of the CRISPR-Cas9 components and their constitutive expression may increase off-target cleavage of similar genomic sequences over time and trigger unwanted immunological response due to the Cas9 bacterial origin.^19^ In fact, stable genome integration of the lentiviral-mediated gene-editing cassette is neither needed nor desirable for clinical application, in that transient expression of both endonuclease and guide RNA is sufficient to attain stable gene editing.

However, human keratinocytes are hard to transfect and genome access turns out to be a major issue. Thus, different transfection methods were attempted to deliver the gene-editing machinery into EBS01 keratinocytes.

Plasmids expressing CRISPR-Cas9 components were initially delivered into cells using commercial lipofectamine reagents, which turned out to be highly inefficient. To implement the transfection efficacy and ensure an optimal editing efficiency, additional electroporation procedures were investigated, all of which were highly toxic for normal human keratinocytes (data not shown).

To overcome these hurdles, EBS01 primary keratinocytes were directly electroporated with a ribonucleoprotein complex (RNP) composed of the SpCas9 endonuclease protein and the guide RNA specific for the mutant allele (sgRNA_del21) (sequence in Table S1).

Following RNP nucleofection, edited EBS01 keratinocytes (eEBS01) underwent further analysis to characterize genotypically and phenotypically the editing impact, always comparing them with a not-transfected EBS01 sample.

Genomic DNA was extracted from sub-confluent eEBS01 and EBS01 cultures and the locus around the 21-bp deletion was amplified by PCR and used to perform Sanger sequencing. The amplicon sequences were analyzed using TIDE analysis (sensitivity > 1%–5%) and validated with next-generation sequencing (NGS).

As shown in Figure 2B, TIDE analysis outlined a strikingly high allele specificity of our tailored gene-editing approach. In eEBS01 cells, the wild-type allele was virtually untouched, whereas the mutant allele displayed a significant amount of insertions or deletions (InDels) abolishing the open reading frame and expression of pathogenic K14. NGS analysis of three independent RNP nucleofections confirmed the remarkable allele specificity (Table S2), with a mutant-allele-specific gene editing greater than 95% (Figure 2C).

Of note, droplet digital PCR (ddPCR) (Figure S2A) and western blot analysis (Figure S2B) showed that editing of the mutant allele restored both *KRT14* mRNA and K14 expression in eEBS01 keratinocytes, as compared to EBS01 cells. Gene-editing specificity was confirmed in an agarose gel analysis, in which PCR products were amplified with oligonucleotides specific for the wild-type (268 bp) and mutant (266 bp) alleles. As shown in Figure 2D, the introduction of several mismatches, after editing and error-prone DNA repair, caused the inability of the forward mutant-allele-specific oligonucleotide (KRT14_seq_del21 primer; see Table S1) to anneal in the edited region, determining the formation of a feebler eEBS01 mutant-allele amplification product. Finally, NGS data were employed to calculate the frequency of deletion, insertion, and substitution in eEBS01 keratinocytes, outlining deletion as the most frequent modification type (75%), whereas insertion and substitution were detected at a lower frequency (19% and 6%, respectively) (Figure 2E). An independent analysis of the NGS data was performed to gain insights into the prevalence of out-of-frame sequences (80% of the total sequences). The most frequent specific rearrangements are illustrated in Figure 2F.

### Off-target analysis supports RNP-complex-mediated editing specificity and safety

To properly address safety issues, a comprehensive analysis assessing potential off-target sites was performed. Genomic DNA purified from sub-confluent EBS01 keratinocytes electroporated with the RNP complex was used to amplify and sequence off-target genomic regions previously identified after lentiviral transduction. TIDE analysis of eEBS01 shows no undesirable changes in any of the six predicted off-target sites, including *PPFIBP1*, thus recovering the unwanted cut chance determined by the genomic lentiviral vector-mediated integration and stable expression of the CRISPR-Cas9 components (Figure S3).

Unbiased genome-wide GUIDE-seq analysis was carried-out and the DNA library was sequenced using Illumina Miseq. The subsequent datasets were analyzed using GUIDE-seq Bioconductor package software. The analysis confirmed preservation of the *KRT14* wild-type allele but outlined nine “putative” off-target sites with a frequency below 3% but above 1% (Table S3). However, the vast majority of the implicated gene regions are either intergenic or intronic. The unique coding region involved is attributed to the *ZNF320* gene, whose expression is anyhow low in basal and superbasal keratinocytes and with a very low GUIDE-seq predicted editing rate. We NGS validated two off-target sites identified in intronic regions (*MIPOL* and *BAIAP*) and in the only coding region (*ZNF320*) detected with GUIDE-seq, for which we obtained appropriate-quality PCR products. NGS showed editing efficiencies of 4.1%, 5.3%, and 6.9% for these respective targets (Table S3; NGS raw data published on GSE246345).

Overall, these data indicate the presence of a small number of potential off-target sites, which have a minimal effect on gene function, underscoring the safety of this non-viral CRISPR-Cas9 approach.

### Editing of KRT14 mutant allele restores functional intermediate filament network in EBS01 keratinocytes

Given the role of K5/K14 pairs in the assembly of keratinocyte intermediate filaments, we investigated whether the unmodified wild-type *KRT14* allele would suffice in restoring normal structural and functional phenotype in edited keratinocytes.

**Figure 3:**
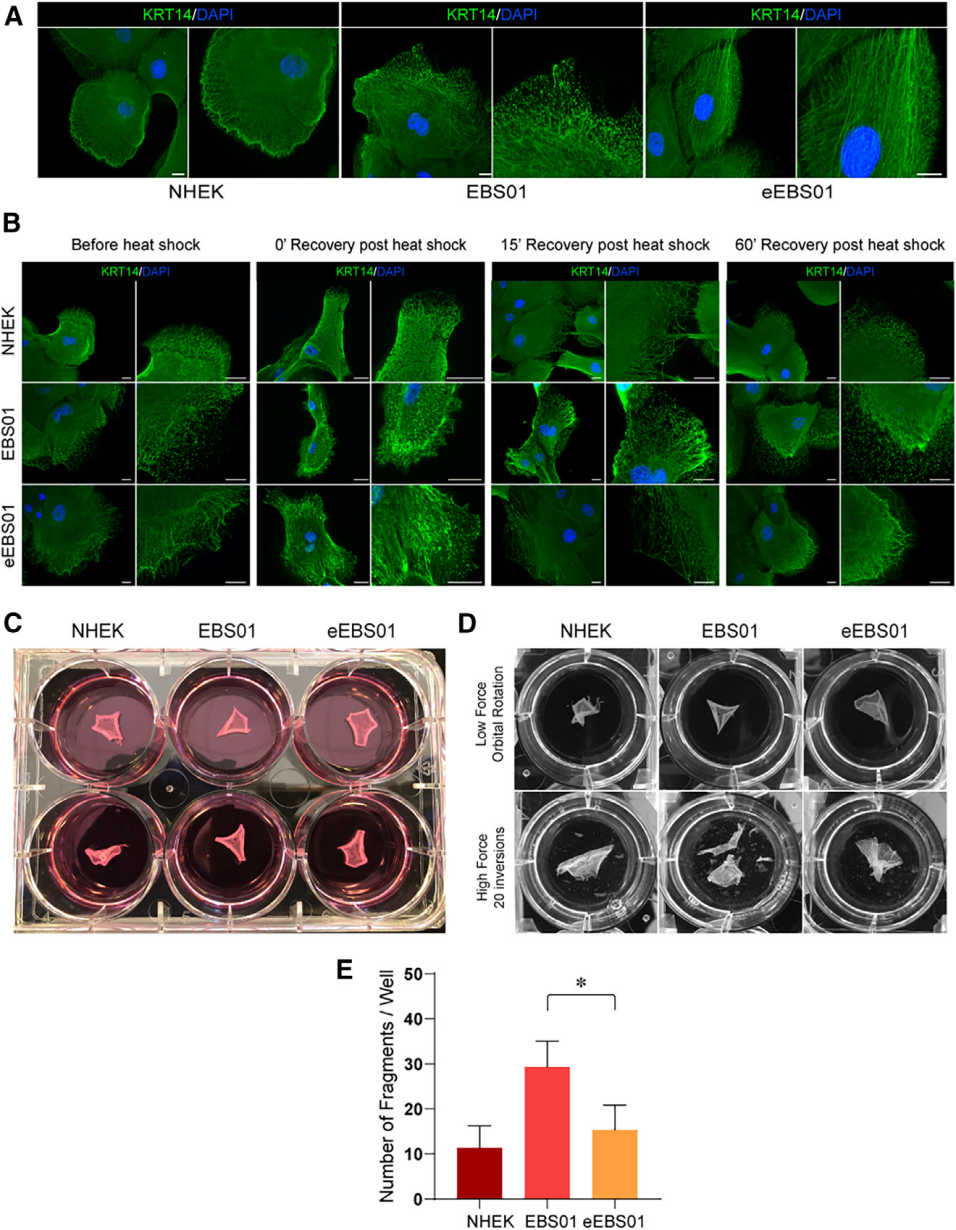
Restoration of intermediate filaments (A) Immunofluorescent staining of K14 intermediate filaments in healthy donor-derived keratinocytes (NHEK) and untreated and treated EBS01 keratinocytes (EBS01 and eEBS01, respectively). Nuclei are stained in blue by DAPI. Scale bar, 20 μm. (B) Immunofluorescent staining of K14 filament before and after heat-shock assay. At time 0 and after 15 min of recovery from heat shock, NEHK and eEBS01 do not show any changes in the organization of cytoplasmatic network, whereas EBS01 keratinocytes display disruption of intermediate filaments also in the perinuclear region. After 60-min recovery, EBS01 intermediate filament aggregates revert to a condition similar to that before the heat shock. Nuclei are stained in blue by DAPI. Scale bar, 20 μm. (C) Dispase-mediated epidermal sheet dissociation assay of NHEK, EBS01, and eEBS01 epidermal cultures before (C) and after (D) mechanical stress. No fragmentation was detected in samples subjected to low-force orbital rotation. In contrast, high-force stress induced fragmentation of EBS01 but not NHEK and eEBS01 sheets (D). (E) Number of fragments derived from each sample has been counted using ImageJ software, and unpaired t test showed a statistically significant p value between EBS01 and eEBS01 number of fragments (N = 3; asterisk indicate p value <0.05).

Healthy donor (normal human epidermal keratinocyte, NHEK) eEBS01 and EBS01 keratinocytes were seeded onto glass coverslips. Immunostained colonies clearly showed that corrected eEBS01 keratinocytes contained a properly functioning intermediate filament network, virtually indistinguishable from that of healthy NHEKs. Keratin filaments were well organized and capable of branching throughout the plasma membrane. In contrast, EBS01 cells displayed a pathologic, roughly fragmentated keratin pattern (Figure 3A). We have analyzed the intermediate filament assembly in 636 NHEK, 722 EBS01 and 1,090 eEBS01 cells. Such analysis is summarized in Figure S4D. The magnified areas shown in Figures S4A–S4C are strictly representative of the several images that we have analyzed in several independent experiments. These data confirmed the efficacy of this gene-editing approach in restoring the EBS altered cellular phenotype. Of note, the ablation of the mutant *KRT14* allele results in *KRT14* haploinsufficiency, which is comparable to the condition characterizing heathy carriers of recessive EBS mutations, who are completely asymptomatic.^20^^,^^21^^,^^22^

Additional functional assays evaluated the regain of mechanical strength in eEBS01 keratinocytes, which is mandatory for a more comprehensive validation of an appropriate functional correction.^23^

Heat-shock assay is one of the easiest and most reproducible tests demonstrating the instability and thermal sensitivity of mutant keratins in EBS cells. The transient increase in thermal energy of the system results in evident depolymerization and impairment in affected keratinocytes’ filament network remodeling, which may render cells vulnerable to cytolysis *in vivo* and support the increased EBS blister formation in warm environments.^24^

To this end, EBS01, eEBS01, and NHEK keratinocyte colonies were submitted to thermal shock. At time zero and 15 min after heat shock, EBS01 keratinocyte’s intermediate filaments showed an increased disruption of keratin filaments, particularly nearby the nuclear region, which was partially recovered approximately 60 min after thermal shock, with aggregates limited to a small portion of the cytoplasm.^25^^,^^26^ Such cytoplasmatic aggregates’ persistence was not observed in NHEK or eEBS01 (Figure 3B).

Since intermediate filaments also play a role in controlling cell mechanical stress, cultured epidermal sheets prepared from NHEK, EBS01, and eEBS01 were detached from the vessel by incubation with Dispase II protease (Figure 3C). EBS01 cultured sheets disintegrated into small pieces when subjected to high inversion force, whereas eEBS01 sheet showed a structural compactness comparable to that of the healthy donor (NHEK) (Figure 3D). The greater eEBS01 mechanical strength was also quantitatively evaluated by counting sheet-derived fragments and confirmed that specific deletion of the mutated *KRT14* allele confers to eEBS01 cells the capability to reassemble proper and resistant cohesive structures (Figure 3E).

### Edited eEBS01 cells revert the disease phenotype in skin equivalents

We have generated human skin equivalents containing dermal and epidermal compartments resembling morphological characteristics of human skin. This was achieved by cultivating keratinocytes on a decellularized human dermal matrix.

**Figure 4:**
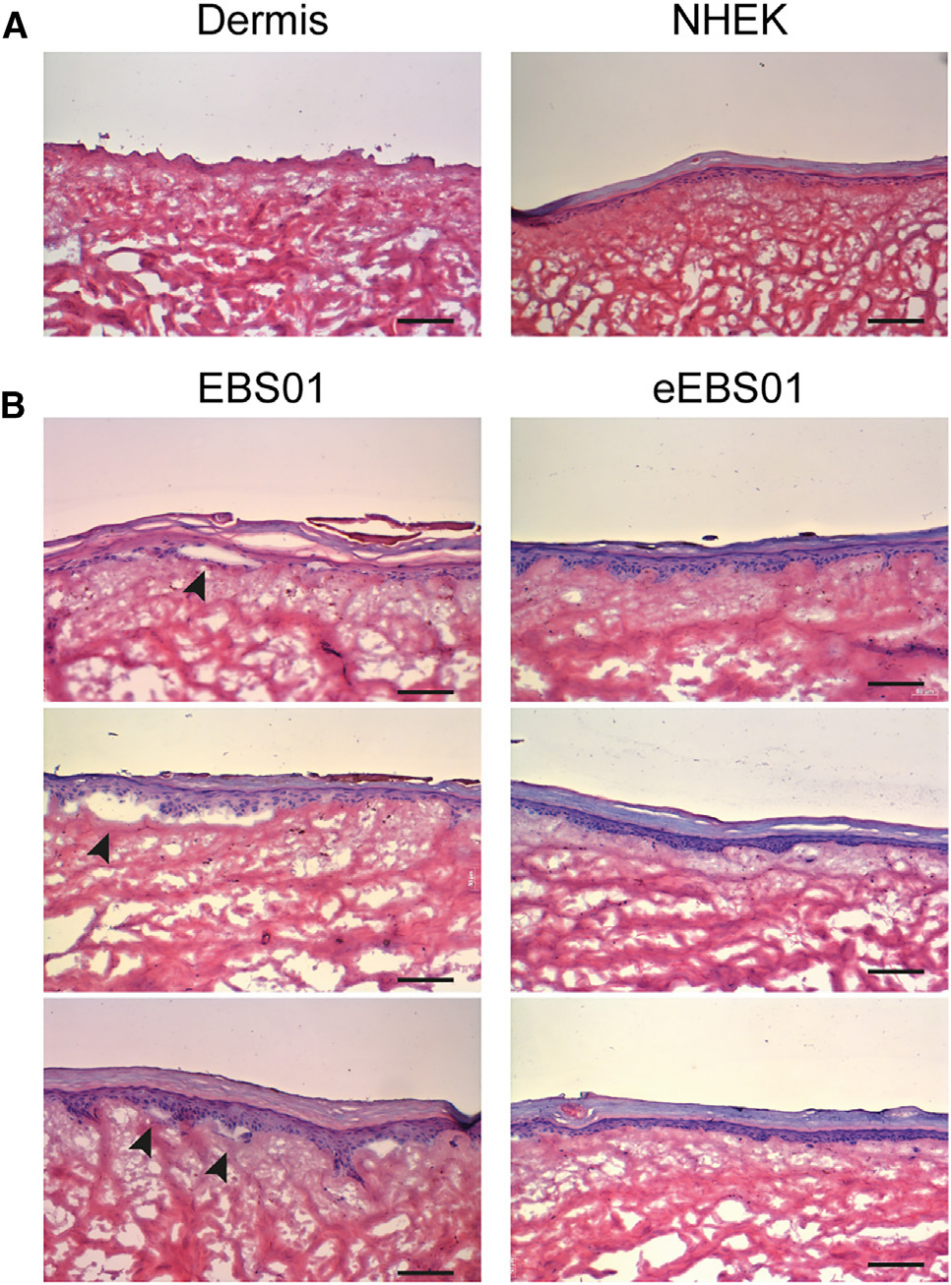
3D skin equivalents (A) Hematoxylin and eosin staining of sections (7-μm thick) of decellularized dermal matrixes (dermis) seeded with NHEKs. (B) The decellularized dermal matrix was seeded with EBS01 or eEBS01 cells. Black arrowheads show blisters originated in the epidermal basal layer only in EBS01 3D cultures. Scale bar, 100 μm.

Figure 4A shows hematoxylin and eosin staining of sections of decellularized dermal matrixes without cells (dermis) and those overlaid by a fully stratified epidermis (NHEK). Figure 4B (left panels) illustrates the decellularized dermal matrix seeded with EBS01 cells. The regenerated epidermis shows the presence of blisters within the epidermal basal layer (arrows). In contrast, no blisters were observed in the epidermis generated by gene-edited eEBS01 keratinocytes (Figure 4B, right panels).

In summary, these results show that the allelic-specific gene editing of mutant *KRT14* restores proper expression of K14 and functional intermediate filaments in primary clonogenic EBS keratinocytes.

### CRISPR-Cas9-mediated gene editing via RNP complex electroporation preserves epidermal stem cells

Epidermal regeneration and repair processes rely on long-lived stem cells producing short-lived transient amplifying (TA) progenitors that eventually give rise to terminally differentiated keratinocytes. Keratinocyte stem cells and TA progenitors are located in the basal layer of all stratified epithelia and generate different clonal types, referred to as holoclones and meroclones/paraclones, respectively.^27^^,^^28^^,^^29^^,^^30^^,^^31^ In view of future clinical applications, the essential feature of any cultured epithelial graft is an adequate number of holoclone-forming cells, which are mandatory for a stable long-term regeneration of all squamous epithelia.^10^^,^^29^^,^^32^^,^^33^^,^^34^ Clonal analysis of EBS01 clonogenic keratinocytes confirmed the presence of each clonal type in the culture (holoclones, meroclones, and paraclones), excluding an impact of this *de novo* mutation on the EBS01-derived keratinocyte stem cell compartment.

**Figure 5:**
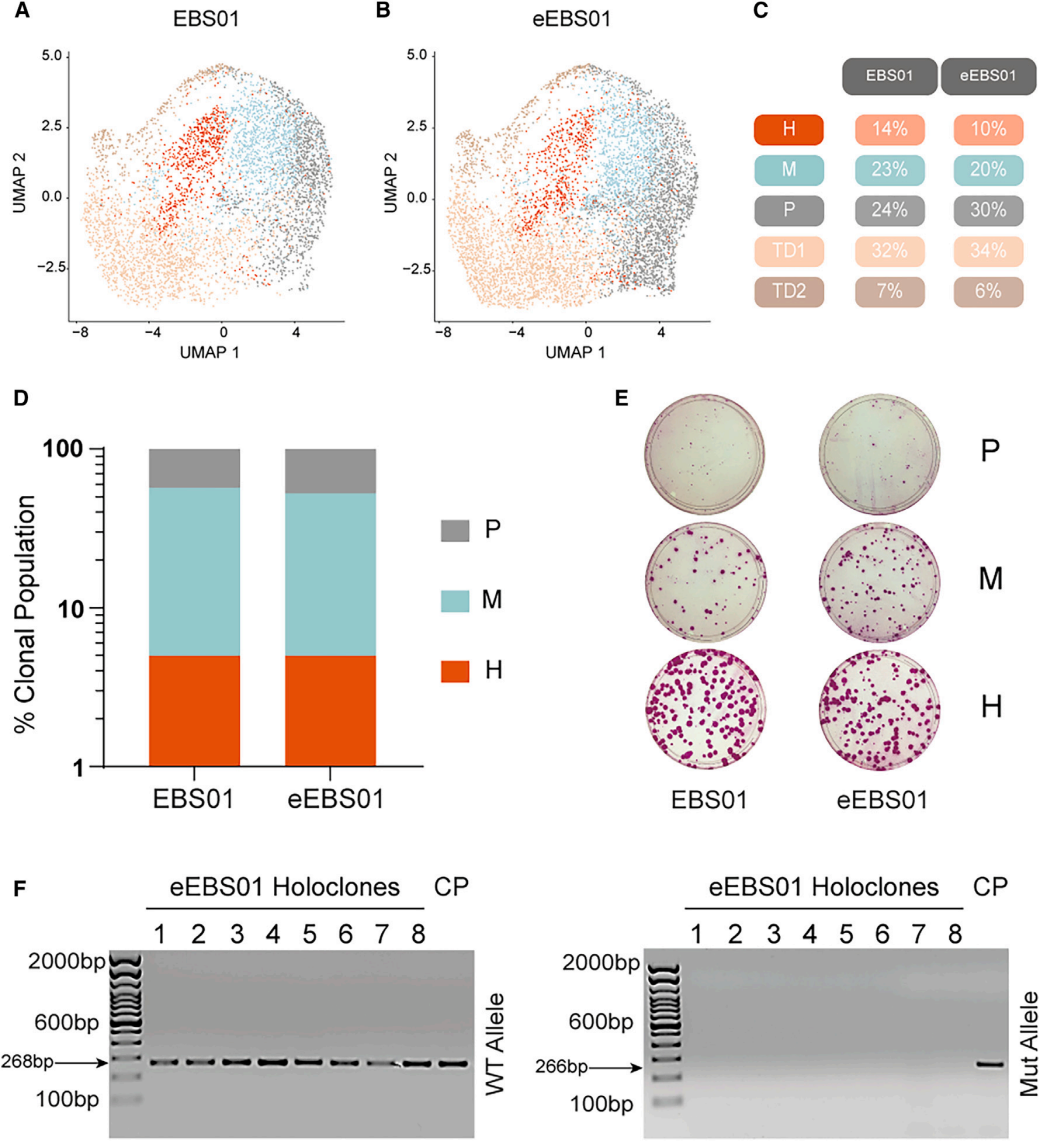
Genetic correction of epidermal stem cells (A and B) Uniform manifold approximation and projection (UMAP) of the scRNA-seq experiment. EBS01 and eEBS01 keratinocytes mass cultures were profiled, integrated, and classified in all their clonogenic and differentiated clusters (H in red, M light blue, P gray, TD1 light brown, TD2 brown). Distribution and extension of the clusters are comparable between the two samples. Feeder-layer-derived fibroblast and low-quality keratinocytes are shown in light gray. (C) Table shows the percentages of cells in each EBS01 and eEBS01 cluster after scRNA-seq analysis. This quantitative analysis demonstrates a comparable percentage between the treated and not-treated samples. (D) Graphical representation of clonal analysis assay results. Clonal population percentages represent the average value of holoclones (H), meroclones (M), and paraclones (P) of two independent experiments (EBS01, holoclones 5%; meroclones 52%, paraclones 43%; eEBS01, holoclones 5%, meroclones 48%, paraclones 47%). As shown, there are no differences in clonal populations between EBS01 and eEBS01 keratinocytes. (E) Representative indicator dishes for EBS01 and eEBS01 holoclones, meroclones, and paraclones. (F) PCR products of holoclone eEBS01’s wild-type and mutant alleles. Wild-type alleles were untouched, whereas all the mutated alleles display editing and lack of primer annealing and amplification. Not-treated EBS01 sample was used as control (CP), in which both the wild-type and mutant allele are present and amplified.

To first investigate a potential impact of gene editing on the distribution of the different keratinocyte clonal types, we took advantage of single-keratinocyte RNA sequencing (RNA-seq) analysis. To this end, we performed transcriptomic profile analysis of EBS01 and eEBS01 keratinocytes following the same pipeline recently published,^28^ obtaining 5,350 and 7,200 cells, respectively. As reported, both samples contained the previously identified five keratinocyte clusters (Figures 5A–5C), three of which expressed clonogenic markers (holoclone, meroclone, and paraclone clusters) and the other two expressing differentiation markers (terminally differentiated 1 and 2 clusters) (Figures S5A and S5B). In particular, the holoclone (stem cell) cluster displayed a “holoclone signature” able to distinguish it from the other clusters (Figures S5A and S5B).

To further investigate whether holoclones were preserved and properly corrected after the gene-editing procedure, two clonal analyses of EBS01 and eEBS01 keratinocytes were performed, as described in section “materials and methods.” The classification of the clonal type confirmed the comparable percentage of holoclones, meroclones, and paraclones in both samples (Figures 5D and 5E). Genomic DNA, analyzed by PCR amplification using primers specific to amplified wild-type and mutant alleles (see Table S1), confirmed that holoclone-forming cells had been edited (Figure 5F).

As shown in Figure S6, both clonogenicity (S6A) and percentage of aborted colonies (S6B) (and growth rate) were comparable in long-term cultures generated by EBS01 and eEBS01 keratinocytes, indicating the absence of clonal selection and/or selective advantages of eEBS01 keratinocytes over the EBS01 cells.

These data demonstrate that the gene-editing procedure was able to preserve and edit the population of epidermal stem cells crucial to a future *ex vivo* gene therapy aimed at full epidermal restoration.

## Discussion

EBS is a rare mechanobullous disease inherited mainly in an autosomal dominant fashion and affecting a few thousands of people worldwide. Mutations affecting *KRT14* account for approximately 30% of the reported cases. Mutant *KRT14* exerts a dominant negative effect on the normal allele, perturbing the basal keratinocyte intermediate filament network and leading to intraepidermal blister formation. Hence, a great portion of EBS patients carrying *KRT14* mutations could potentially benefit from the correction of the genetic defect.^35^

Gene replacement strategies, whereby a functional copy of the defective gene is introduced in the genome of clonogenic keratinocytes, has been successfully exploited in other forms of EB, such as the *LAMB3*-dependent JEB and recessive DEB (RDEB), which are recessively inherited.^6^^,^^8^^,^^10^^,^^11^^,^^12^^,^^13^ However, gene replacement is not appropriate for the treatment of dominantly inherited genetic diseases, which instead require either the selective disruption of the mutated allele or the precise editing of the specific mutation.^36^^,^^37^ An appropriately designed gene-editing machinery would allow us to discriminate and inactivate, via the error-prone NHEJ pathway, only the mutant allele, leaving the wild-type counterpart functionally intact. Gene editing has been employed to permanently repair both dominant^38^^,^^39^^,^^40^^,^^41^ and recessive^42^^,^^43^^,^^44^ mutations related to EB using knockout and homologous recombination techniques. The end-joining pathways, often harnessed for gene knockout, represent the most efficient repair mechanisms for DSBs.^45^ Consequently, gene knockout is the most effective form of gene editing.

This work presents the first evidence of therapeutically relevant allele-specific genetic correction in primary cultures of EBS-derived epidermal stem cells. A CRISPR-Cas9-based allele-specific editing platform using a specific single guide-RNA (sgRNA) successfully edited a *de novo* c.475/495del21 mutation within exon 1 of the *KRT14* gene in a dominant form of EBS. Our tailored approach was highly effective in disrupting the mutant *KRT14* allele on EBS01 primary keratinocytes (editing efficiency greater than 95%) with no editing on the wild-type allele. To ensure transient expression of editing machinery suitable for clinical purposes, the sgRNA-Cas9 RNP complex has been directly delivered to EBS01 keratinocytes. Since indels generation at off-target sites still pose a risk to the use of engineered nucleases, we also demonstrated a low occurrence of non-specific CRISPR-Cas9-mediated cleavages through unbiased GUIDE-seq analysis.

Thus, this approach succeeded in abolishing the mutant *KRT14* allele expression and the pathogenic keratin almost entirely and fully restored a functional intermediate filament network. More importantly, we provide evidence that the long-lived self-renewing stem cells have been targeted and corrected by the gene-editing machinery without any cytotoxicity, thus maintaining the ability to regenerate a virtually indistinguishable functional epidermis.

The selection of a gene-editing tool for allele-specific genetic correction depends on factors such as the specific genetic mutation, the delivery method, the desired level of precision, and (perhaps more importantly) the cell type, mainly when specific somatic stem cells need to be targeted. In the case of EBS01 cells, the deletion of 21 bp (c.475/495del21) within exon 1 of *KRT14* led us to design an sgRNA tailored to specifically target the mutant allele. We opted for a gene-editing approach utilizing SpCas9, known for its high cutting efficiency. We introduced the sgRNA and SpCas9 as an RNP complex that overcomes many of the challenges associate with mRNA delivery, as the translation steps and the folding of the Cas protein. The RNP complex is immediately active as it is fully developed. Emerging gene-editing tools may offer more precise approaches than traditional methods, but often have an efficiency not sufficient to fully tackle a specific population of epidermal stem cells, which represent a small percentage of clonogenic keratinocytes. Promising gene-editing tools for allele-specific genetic correction include prime editing,^46^^,^^47^ base editing,^48^^,^^49^ and Cpf1/Cas12-based editing,^50^^,^^51^^,^^52^ which provides versatility by targeting distinct DNA sequences, widening the scope of targetable genetic mutations. These tools hold great therapeutic promise due to their precision and reduced off-target effects.

In the context of clinical translation and safety of gene-editing strategies, a key aspect is the analysis of off-target effects. As sequencing technologies and data analysis tools continue to advance, there may be, in the near future, more cost-effective and high-throughput options for off-target analysis than whole-genome sequencing.

Our study provides a clear demonstration of the efficacy and potential safety of an allele-specific CRISPR-based gene-editing approach, which we envision to further translate into a long-lasting decisive clinical treatment for patients suffering from EBS and possibly other related skin-blistering diseases. Although we did not observe abnormal clonal expansion, clinical translation would require additional efforts to determine the optimal RNP dosage to achieve maximal on-target efficiency and minimal off-target impact and thoroughly verify the absence of genotoxicity and genomic instabilities.

## Materials and methods

### Patient, clinical data, and treatments

The EBS patient (EBS01) displayed skin lesions to exclusively the acral regions and no mucosal blisters or erosions were ever reported either by him or his parents. Clinical description and genetic counseling with genetic analysis of the family are given in the “supplemental materials and methods” section.

### Structural modeling

The structure predictions were performed in a standalone platform of AlphaFold2 and AlphaFold-Multimer^15^ as implemented in ColabFold, which was set up on a local computer with a Linux operating system and accelerated with two NVIDIA GeForce RTX 2080 Ti GPU. The Template mode using PDB 3TNU ^16^ was used for this purpose. The other parameters were kept at their default values.

### Primary human cell culture from the EBS patient

A skin biopsy was collected from the EBS01 patient after obtaining informed consent. Briefly, skin biopsy was minced and treated with 0.05% trypsin/0.01% EDTA for 4 h at 37°C. Every 30 min, keratinocytes were collected, plated (2.5–3 × 10^4^ cells/cm^2^) on lethally irradiated 3T3-J2-Y cells, and grown at 37°C, 5% CO_2_ in humidified atmosphere in KGM (Keratinocyte Growth Medium): Dulbecco’s modified Eagle’s medium (DMEM) and Ham’s F12 medium (2:1 mixture) containing fetal bovine serum (FBS) (10%), penicillin-streptomycin (50 IU/mL), glutamine (4 mM), adenine (0.18 mM), insulin (5 mg/mL), cholera toxin (0.1 nM), hydrocortisone (1.1 mM), triiodothyronine (lithyronine sodium, 2 nM), and epidermal growth factor (EGF, 10 ng/mL). When sub-confluent, cell cultures were serially propagated.

### 3T3-J2 cell line

The mouse 3T3-J2 cells were a gift from Professor Howard Green, Harvard Medical School (Boston, MA, USA).^53^ Fibroblasts were cultivated in DMEM supplemented with 10% γ-irradiated donor adult bovine serum, penicillin-streptomycin (50 IU/mL), and glutamine (4 mM). BioNTech (Germany) produce a GMP clinical-grade 3T3-J2 cell bank that has been authorized for clinical use by national and European regulatory authorities.

### RNP complex formation and nucleofection

The synthetic guide RNA was designed straddling both the terminal sides of the c475/495del21 mutation (5′-GCTGAGGTTCAAGACCATTG-3′) and directly flanking an AGG PAM. It was modified to drive the maximum editing efficiency (Invitrogen, #A35514; Table S1) and was mixed in a 1.1:1 molar ratio with the Cas9 protein (Alt-R S.p. Cas9 Nuclease V3, IDT, #1081058). Then 5 × 10^5^ keratinocytes were resuspended in 100 μL of primary cell nucleofection solution (P3 Primary Cell 4D-Nucleofector Kit, Lonza, #LOV4XP3024) mixed with the RNP complex solution and 4 μM Cas9 electroporation enhancer (Alt-R Cas9 Electroporation Enhancer, IDT, #1075915). Cells were electroporated using a 4D-Nucleofector (4D-Nucleofector Core Unit, Lonza, #AAF-1001B; 4D-Nucleofector X Unit, Lonza, #AAF-1001X) using the program DS-138.

### Editing analysis by sequence decomposition (TIDE)

eEBS01 and EBS01 keratinocytes genomic DNA was isolated using the QIAamp DNA Mini Kit (QIAGEN, #51304). A 500-bp region around the eEBS01 and EBS01 genomic target site was amplified by PCR (primers in Table S1). PCR amplicons were subjected to conventional Sanger sequencing. The resulting sequence trace files were uploaded on the TIDE web tool with the guide RNA sequence as input.

### PCR and allele characterization

Screening of the allele pattern, in eEBS01 and EBS01 keratinocytes, was done by PCR. The KRT14_seq_del21 forward primer was specific only for the mutant allele in EBS01 keratinocyte, and KRT14_seq_wt forward primer was specific only for the wild-type allele. Reverse primer was specific for a sequence common to both alleles (Table S1).

### OFF-target analysis

CRISPR-Cas9 online predictors were used to identify the genomic regions that may present the greatest probability of off-target cuttings. Off-target probability was evaluated on the basis of mismatch numbers, and the genomic loci of the most probable *in silico* off-target sites were sequenced and analyzed. The resulting sequence trace files were uploaded on the TIDE web tool with the guide RNA sequence as input.

### NGS analysis

The region near the target site was amplified by specific PCR primers with sequence adaptor (Table S1, KRT14ex1_NGS and KRT14intr1_NGS) and 25 μL of purified amplicon was used for NGS analysis using the Illumina sequencing platform. The clipping of reads was performed using Trimmomatic (v 0.36) and paired-end reads were merged using software FLASH2 (v 2.2.00) to obtain a single, longer read that covers the full target region. The processed reads were mapped, using BMWA MEM (v 0.7.15), to the reference sequence (the wild-type *KRT14* exon1 sequence) with default alignment parameters. Only high-quality, merged, on-target reads were considered for further processing. Finally, the identification and quantification of allelic sequences using CRISPResso (v 1.0.13) occurred. The NGS raw data are available in Gene Expression Omnibus with accession number GSE246345.

### Clonal analysis

Sub-confluent keratinocytes mass cultures were trypsinized and 0.5–1 cell was plated into each well of a 96-well plate after serial dilution. After 7 days of cultivation, single clones were identified under an inverted microscope and treated with 0.05% trypsin and 0.01% EDTA at 37° for 15–20 min. One-quarter of the clone was plated onto a 100-mm indicator dish, cultivated for 12 days, and stained with rhodamine B for the classification of clonal type. The remaining three-quarters was sub-cultivated on an adequate plastic support and used for further analyses.^34^

### Immunofluorescence

Keratinocytes were plated at 2,500 cells/well onto glass coverslips. After the formation of small colonies, cells were fixed with ice-cold methanol-acetone (1:1) at −20°C for 10 min. Cells were permeabilized with PBS/Triton 0.5% for 15 min. Blocking solution (BSA 5%, 0.3% Triton in 1× PBS) was added for 30 min at 37°C and sections were incubated with primary and secondary antibody (Table S1). Cell nuclei were stained with DAPI. Fluorescence signals were monitored under a Zeiss Axio Imager A.1 Manual Operation Fluorescence Microscope with EC Plan-Neofluar 40×/0.75 objective.

### GUIDE-seq analysis

The 5 × 10^5^ primary EBS01 keratinocytes were nucleofected (as described in section “RNP complex formation and nucleofection”) with the RNP complex and 40 nM annealed double-stranded oligodeoxynucleotide (dsODN). Treated keratinocytes were then plated (4–6 × 10^3^ cells/cm^2^) on lethally irradiated 3T3-J2 cells and cultured until sub-confluence. Keratinocytes’ genomic DNA was extracted and 14 μg was sent to Creative Biogene for GUIDE-seq library preparation and sequencing in order to identify CRISPR RNA-guided nuclease (RGN)-dependent and -independent genomic breakpoint “hotspots.” DNA library was sequenced using Illumina Miseq. The subsequent datasets were analyzed using the *GUIDEseq* Bioconductor package software.

### Encapsulation with 10X Genomics chromium system and bioinformatic analysis on single-cell RNA-seq data

Fully confluent keratinocytes were detached and cells were accurately resuspended to obtain a single-cell suspension. About 10,000 cells of each eEBS01 and EBS01 sample were loaded into two channels of the Chromium Chip B using the Single Cell reagent kit v3.1 (10X Genomics) for gel bead emulsion generation. Following capture and lysis, cDNA was synthesized and amplified. Fifty nanograms of the amplified cDNA were then used for each sample to construct Illumina sequencing libraries. Sequencing was performed on the NextSeq550 Illumina sequencing platform following the 10X Genomics instruction for read generation, reaching at least 50,000 reads as mean reads per cell.

For the bioinformatic analysis, the Cell Ranger pipeline (version 3.1.0) was used to generate FASTQ files, to align reads to the reference transcriptome (GRCh38) and to calculate UMI (unique molecular identifier) counts from the mapped reads. Expression data were imported in R version 3.6.3 and analyzed using Seurat^54^ (version 3.1.5) R package. Cells were classified using an annotated single-cell RNA-seq (scRNA-seq) dataset of human keratinocytes^28^ as reference and the FindTransferAnchors and TransferData functions in Seurat with default parameters. We assessed the quality of the assigned labels monitoring the expression of known markers. Expression data are available in Gene Expression Omnibus with accession number GSE246345.

### Heat-shock assay

Keratinocytes were plated at 10,000 cells/well onto glass coverslips. Cell culture medium was replaced with KGM medium at 43°C and the well plate was immediately placed in a water bath set at 47°C. After 15 min of heat stress, the medium was immediately replaced with fresh KGM medium at 37°C and the cells were allowed to recover in the incubator at 37°C, 5% CO_2_ in humidified atmosphere. Coverslips were removed at 15-min intervals thereafter and immunostained.

### Dispase-based keratinocyte dissociation assay

4–6 × 10^3^ keratinocytes/cm^2^ were plated on lethally irradiated 3T3-J2 cells onto a six-well plate and cultured. After 20 days, the epidermal sheets were washed with grafting wash and incubated with Dispase II (Roche, #0494207801), 2.5U/mL diluted in PBS, for 1 h at 37°C. After detachment, one sheet was subjected to low-force stress with orbital rotation (200 rpm) for 5 min at 37°C and the other monolayer was transferred in a 15-mL Falcon tube with 5 mL of 1× PBS and exposed to high mechanical stress by 20–50 inversions. Fragments count was performed with ImageJ.

### Decellularized dermal matrixes preparation, cryopreservation, and sectioning

Decellularized human dermal matrixes were obtained using human skin samples from surgical waste (abdominoplasty or mammoplasty). Briefly, skin biopsies were sectioned in fragments of approximately 1.5 cm^2^, immersed in sterile PBS at 60°C for 30 s under constant stirring, and then placed in sterile cold PBS for 1 min. The epidermis was then mechanically detached from the dermis using forceps. After decellularization, samples were rinsed in KGM for 24 h. The following day, the decellularized dermal matrixes were seeded with primary human keratinocytes (1 × 10^5^ cells per scaffold) onto lethally irradiated 3T3-J2 cells (5 × 10^4^ cells per scaffold) in KGM. After 10 days in submerged culture, the medium was carefully removed and the samples were gently moved in Millicell cell culture (Merk) and were further cultured for 20–24 days in air-liquid interface (ALI) conditions to induce epidermal differentiation. Then 3D human skin equivalents were dehydrated in a sucrose gradient 0.9 and 2 M for 30 min respectively at room temperature (RT), embedded in Killik-OCT cryostat embedding medium (Bio-Optica), and frozen. The 7-μm sections of embedded skin equivalents were obtained with a histological cryomicrotome (Leica CM1850 UV).

### Hematoxylin and eosin staining

Hematoxylin and eosin staining was performed on 7-μm cryosections of decellularized dermal matrixes (Harris hematoxylin for 1 min, running tap water for 1 min, eosin Y 50% in ethanol for 30 s, 95% ethanol for 1 min, 100% ethanol for 1 min, two rinses in fresh 100% ethanol for 1 min each) and observed with a Zeiss Microscope Axio Imager M2 with an EC Plan-Neofluar 10×/0.3 M27 air objective.

## Data and code availability

Data availability sequencing data have been deposited to Gene Expression Omnibus with accession number GSE246345. We declare that the data supporting the findings of this study are available within the paper and its supplemental information files or from the authors upon request.

## Supplemental information

Document S1. Supplemental materials and methods, Figures S1–S6 and Tables S1–S3

Document S2. Article plus supplemental information

## References

[1] Coulombe P.A., Kerns M.L., Fuchs E. (2009). Epidermolysis bullosa simplex: a paradigm for disorders of tissue fragility. J. Clin. Invest..

[2] Chamcheu J.C., Siddiqui I.A., Syed D.N., Adhami V.M., Liovic M., Mukhtar H. (2011). Keratin gene mutations in disorders of human skin and its appendages. Arch. Biochem. Biophys..

[3] Fine J.-D., Bruckner-Tuderman L., Eady R.A.J., Bauer E.A., Bauer J.W., Has C., Heagerty A., Hintner H., Hovnanian A., Jonkman M.F. (2014). Inherited epidermolysis bullosa: Updated recommendations on diagnosis and classification. J. Am. Acad. Dermatol..

[4] Fine J.-D., Eady R.A.J., Bauer E.A., Bauer J.W., Bruckner-Tuderman L., Heagerty A., Hintner H., Hovnanian A., Jonkman M.F., Leigh I. (2008). The classification of inherited epidermolysis bullosa (EB): Report of the Third International Consensus Meeting on Diagnosis and Classification of EB. J. Am. Acad. Dermatol..

[5] Sprecher E. (2010). Epidermolysis Bullosa Simplex. Dermatol. Clin..

[6] Bauer J.W., Koller J., Murauer E.M., De Rosa L., Enzo E., Carulli S., Bondanza S., Recchia A., Muss W., Diem A. (2017). Closure of a Large Chronic Wound through Transplantation of Gene-Corrected Epidermal Stem Cells. J. Invest. Dermatol..

[7] De Rosa L., Enzo E., Zardi G., Bodemer C., Magnoni C., Schneider H., De Luca M. (2021). Hologene 5: A Phase II/III Clinical Trial of Combined Cell and Gene Therapy of Junctional Epidermolysis Bullosa. Front. Genet..

[8] De Rosa L., Carulli S., Cocchiarella F., Quaglino D., Enzo E., Franchini E., Giannetti A., De Santis G., Recchia A., Pellegrini G., De Luca M. (2014). Long-Term Stability and Safety of Transgenic Cultured Epidermal Stem Cells in Gene Therapy of Junctional Epidermolysis Bullosa. Stem Cell Rep.

[9] Eichstadt S., Tang J.Y., Solis D.C., Siprashvili Z., Marinkovich M.P., Whitehead N., Schu M., Fang F., Erickson S.W., Ritchey M.E. (2019). From Clinical Phenotype to Genotypic Modelling: Incidence and Prevalence of Recessive Dystrophic Epidermolysis Bullosa (RDEB). Clin. Cosmet. Investig. Dermatol..

[10] Hirsch T., Rothoeft T., Teig N., Bauer J.W., Pellegrini G., De Rosa L., Scaglione D., Reichelt J., Klausegger A., Kneisz D. (2017). Regeneration of the entire human epidermis using transgenic stem cells. Nature.

[11] Kueckelhaus M., Rothoeft T., De Rosa L., Yeni B., Ohmann T., Maier C., Eitner L., Metze D., Losi L., Secone Seconetti A. (2021). Transgenic Epidermal Cultures for Junctional Epidermolysis Bullosa — 5-Year Outcomes. N. Engl. J. Med..

[12] Mavilio F., Pellegrini G., Ferrari S., Di Nunzio F., Di Iorio E., Recchia A., Maruggi G., Ferrari G., Provasi E., Bonini C. (2006). Correction of junctional epidermolysis bullosa by transplantation of genetically modified epidermal stem cells. Nat. Med..

[13] Siprashvili Z., Nguyen N.T., Gorell E.S., Loutit K., Khuu P., Furukawa L.K., Lorenz H.P., Leung T.H., Keene D.R., Rieger K.E. (2016). Safety and Wound Outcomes Following Genetically Corrected Autologous Epidermal Grafts in Patients With Recessive Dystrophic Epidermolysis Bullosa. JAMA.

[14] Chakravarti S., Enzo E., Rocha Monteiro de Barros M., Maffezzoni M.B.R., Pellegrini G. (2022). Genetic Disorders of the Extracellular Matrix: From Cell and Gene Therapy to Future Applications in Regenerative Medicine. Annu. Rev. Genomics Hum. Genet..

[15] Jumper J., Evans R., Pritzel A., Green T., Figurnov M., Ronneberger O., Tunyasuvunakool K., Bates R., Žídek A., Potapenko A. (2021). Highly accurate protein structure prediction with AlphaFold. Nature.

[16] Lee C.-H., Kim M.-S., Chung B.M., Leahy D.J., Coulombe P.A. (2012). Structural basis for heteromeric assembly and perinuclear organization of keratin filaments. Nat. Struct. Mol. Biol..

[17] Rugg E.L., Morley S.M., Smith F.J., Boxer M., Tidman M.J., Navsaria H., Leigh I.M., Lane E.B. (1993). Missing links: Weber–Cockayne keratin mutations implicate the L12 linker domain in effective cytoskeleton function. Nat. Genet..

[18] Parry D.A.D., Marekov L.N., Steinert P.M., Smith T.A. (2002). A Role for the 1A and L1 Rod Domain Segments in Head Domain Organization and Function of Intermediate Filaments: Structural Analysis of Trichocyte Keratin. J. Struct. Biol..

[19] Charlesworth C.T., Deshpande P.S., Dever D.P., Camarena J., Lemgart V.T., Cromer M.K., Vakulskas C.A., Collingwood M.A., Zhang L., Bode N.M. (2019). Identification of preexisting adaptive immunity to Cas9 proteins in humans. Nat. Med..

[20] Batta K., Rugg E.L., Wilson N.J., West N., Goodyear H., Lane E.B., Gratian M., Dopping-Hepenstal P., Moss C., Eady R.A.J. (2000). A keratin 14 `knockout’ mutation in recessive epidermolysis bullosa simplex resulting in less severe disease. Br. J. Dermatol..

[21] Yiasemides E., Trisnowati N., Su J., Dang N., Klingberg S., Marr P., Melbourne W., Tran K., Chow C.W., Orchard D. (2008). Clinical heterogeneity in recessive epidermolysis bullosa due to mutations in the keratin 14 gene, *KRT14*. Clin. Exp. Dermatol..

[22] Sørensen C.B., Andresen B.S., Jensen U.B., Jensen T.G., Jensen P.K.A., Gregersen N., Bolund L. (2003). Functional testing of keratin 14 mutant proteins associated with the three major subtypes of epidermolysis bullosa simplex. Exp. Dermatol..

[23] Tan T.S., Ng Y.Z., Badowski C., Dang T., Common J.E.A., Lacina L., Szeverényi I., Lane E.B. (2016). Methods in Enzymology.

[24] Morley S.M., Dundas S.R., James J.L., Gupta T., Brown R.A., Sexton C.J., Navsaria H.A., Leigh I.M., Lane E.B. (1995). Temperature sensitivity of the keratin cytoskeleton and delayed spreading of keratinocyte lines derived from EBS patients. J. Cell Sci..

[25] Coulombe P.A., Hutton M.E., Letai A., Hebert A., Paller A.S., Fuchs E. (1991). Point mutations in human keratin 14 genes of epidermolysis bullosa simplex patients: Genetic and functional analyses. Cell.

[26] Kitajima Y., Inoue S., Yaoita H. (1989). Abnormal organization of keratin intermediate filaments in cultured keratinocytes of epidermolysis bullosa simplex. Arch. Dermatol. Res..

[27] Barrandon Y., Green H. (1987). Three clonal types of keratinocyte with different capacities for multiplication. Proc. Natl. Acad. Sci..

[28] Enzo E., Secone Seconetti A., Forcato M., Tenedini E., Polito M.P., Sala I., Carulli S., Contin R., Peano C., Tagliafico E. (2021). Single-keratinocyte transcriptomic analyses identify different clonal types and proliferative potential mediated by FOXM1 in human epidermal stem cells. Nat. Commun..

[29] Pellegrini G., Golisano O., Paterna P., Lambiase A., Bonini S., Rama P., De Luca M. (1999). Location and Clonal Analysis of Stem Cells and Their Differentiated Progeny in the Human Ocular Surface. J. Cell Biol..

[30] Rochat A., Kobayashi K., Barrandon Y. (1994). Location of stem cells of human hair follicles by clonal analysis. Cell.

[31] Polito M.P., Marini G., Palamenghi M., Enzo E. (2023). Decoding the Human Epidermal Complexity at Single-Cell Resolution. Int. J. Mol. Sci..

[32] De Rosa L., Enzo E., Palamenghi M., Sercia L., De Luca M. (2023). Stairways to Advanced Therapies for Epidermolysis Bullosa. Cold Spring Harb. Perspect. Biol..

[33] Rama P., Matuska S., Paganoni G., Spinelli A., De Luca M., Pellegrini G. (2010). Limbal Stem-Cell Therapy and Long-Term Corneal Regeneration. N. Engl. J. Med..

[34] Enzo E., Cattaneo C., Consiglio F., Polito M.P., Bondanza S., De Luca M. (2022). Methods in Cell Biology.

[35] Bolling M.C., Lemmink H.H., Jansen G.H.L., Jonkman M.F. (2011). Mutations in KRT5 and KRT14 cause epidermolysis bullosa simplex in 75% of the patients: KRT5 and KRT14 mutations in 75% of EBS patients. Br. J. Dermatol..

[36] György B., Nist-Lund C., Pan B., Asai Y., Karavitaki K.D., Kleinstiver B.P., Garcia S.P., Zaborowski M.P., Solanes P., Spataro S. (2019). Allele-specific gene editing prevents deafness in a model of dominant progressive hearing loss. Nat. Med..

[37] Latella M.C., Di Salvo M.T., Cocchiarella F., Benati D., Grisendi G., Comitato A., Marigo V., Recchia A. (2016). In vivo Editing of the Human Mutant Rhodopsin Gene by Electroporation of Plasmid-based CRISPR/Cas9 in the Mouse Retina. Mol. Ther. Nucleic Acids.

[38] Kocher T., Peking P., Klausegger A., Murauer E.M., Hofbauer J.P., Wally V., Lettner T., Hainzl S., Ablinger M., Bauer J.W. (2017). Cut and Paste: Efficient Homology-Directed Repair of a Dominant Negative KRT14 Mutation via CRISPR/Cas9 Nickases. Mol. Ther..

[39] March O.P., Lettner T., Klausegger A., Ablinger M., Kocher T., Hainzl S., Peking P., Lackner N., Rajan N., Hofbauer J.P. (2019). Gene Editing–Mediated Disruption of Epidermolytic Ichthyosis–Associated KRT10 Alleles Restores Filament Stability in Keratinocytes. J. Invest. Dermatol..

[40] Luan X.-R., Chen X.-L., Tang Y.-X., Zhang J.-Y., Gao X., Ke H.-P., Lin Z.-Y., Zhang X.-N. (2018). CRISPR/Cas9-Mediated Treatment Ameliorates the Phenotype of the Epidermolytic Palmoplantar Keratoderma-like Mouse. Mol. Ther. Nucleic Acids.

[41] Aushev M., Koller U., Mussolino C., Cathomen T., Reichelt J. (2017). Traceless Targeting and Isolation of Gene-Edited Immortalized Keratinocytes from Epidermolysis Bullosa Simplex Patients. Mol. Ther. Methods Clin. Dev..

[42] Webber B.R., Osborn M.J., McElroy A.N., Twaroski K., Lonetree C.L., DeFeo A.P., Xia L., Eide C., Lees C.J., McElmurry R.T. (2016). CRISPR/Cas9-based genetic correction for recessive dystrophic epidermolysis bullosa. Npj Regen. Med..

[43] Hainzl S., Peking P., Kocher T., Murauer E.M., Larcher F., Del Rio M., Duarte B., Steiner M., Klausegger A., Bauer J.W. (2017). COL7A1 Editing via CRISPR/Cas9 in Recessive Dystrophic Epidermolysis Bullosa. Mol. Ther..

[44] Wu W., Lu Z., Li F., Wang W., Qian N., Duan J., Zhang Y., Wang F., Chen T. (2017). Efficient *in vivo* gene editing using ribonucleoproteins in skin stem cells of recessive dystrophic epidermolysis bullosa mouse model. Proc. Natl. Acad. Sci..

[45] Danner E., Bashir S., Yumlu S., Wurst W., Wefers B., Kühn R. (2017). Control of gene editing by manipulation of DNA repair mechanisms. Mamm. Genome.

[46] Nelson J.W., Randolph P.B., Shen S.P., Everette K.A., Chen P.J., Anzalone A.V., An M., Newby G.A., Chen J.C., Hsu A., Liu D.R. (2022). Engineered pegRNAs improve prime editing efficiency. Nat. Biotechnol..

[47] Petri K., Zhang W., Ma J., Schmidts A., Lee H., Horng J.E., Kim D.Y., Kurt I.C., Clement K., Hsu J.Y. (2022). CRISPR prime editing with ribonucleoprotein complexes in zebrafish and primary human cells. Nat. Biotechnol..

[48] Huang T.P., Newby G.A., Liu D.R. (2021). Precision genome editing using cytosine and adenine base editors in mammalian cells. Nat. Protoc..

[49] Antoniou P., Hardouin G., Martinucci P., Frati G., Felix T., Chalumeau A., Fontana L., Martin J., Masson C., Brusson M. (2022). Base-editing-mediated dissection of a γ-globin cis-regulatory element for the therapeutic reactivation of fetal hemoglobin expression. Nat. Commun..

[50] Kleinstiver B.P., Sousa A.A., Walton R.T., Tak Y.E., Hsu J.Y., Clement K., Welch M.M., Horng J.E., Malagon-Lopez J., Scarfò I. (2019). Engineered CRISPR–Cas12a variants with increased activities and improved targeting ranges for gene, epigenetic and base editing. Nat. Biotechnol..

[51] Strecker J., Jones S., Koopal B., Schmid-Burgk J., Zetsche B., Gao L., Makarova K.S., Koonin E.V., Zhang F. (2019). Engineering of CRISPR-Cas12b for human genome editing. Nat. Commun..

[52] McMahon M.A., Prakash T.P., Cleveland D.W., Bennett C.F., Rahdar M. (2018). Chemically Modified Cpf1-CRISPR RNAs Mediate Efficient Genome Editing in Mammalian Cells. Mol. Ther..

[53] Todaro G.J., Green H., Goldberg B.D. (1964). TRANSFORMATION OF PROPERTIES OF AN ESTABLISHED CELL LINE BY SV40 AND POLYOMA VIRUS. Proc. Natl. Acad. Sci..

[54] Stuart T., Butler A., Hoffman P., Hafemeister C., Papalexi E., Mauck W.M., Hao Y., Stoeckius M., Smibert P., Satija R. (2019). Comprehensive Integration of Single-Cell Data. Cell.

