# Supplementary material for: Allele-specific CRISPR-Cas9 editing of dominant epidermolysis bullosa simplex in human epidermal stem cells
Source: Mol Ther. 2023 Dec 5;32(2):372–83. doi: 10.1016/j.ymthe.2023.11.027 (PMC10861943; doi:10.1016/j.ymthe.2023.11.027)
Supplement: Document S1. Supplemental materials and methods, Figures S1–S6 and Tables S1–S3 [file mmc1.pdf]

YMTHE, Volume *32*

Supplemental Information

Allele-specific CRISPR-Cas9 editing of dominant epidermolysis bullosa simplex in human epidermal stem cells

C. Cattaneo, E. Enzo, L. De Rosa, L. Sercia, F. Consiglio, M. Forcato, S. Bicciato, A. Paiardini, G. Basso, E. Tagliafico, A. Paganelli, C. Fiorentini, C. Magnoni, M.C. Latella, and M. De Luca

Supplemental Material

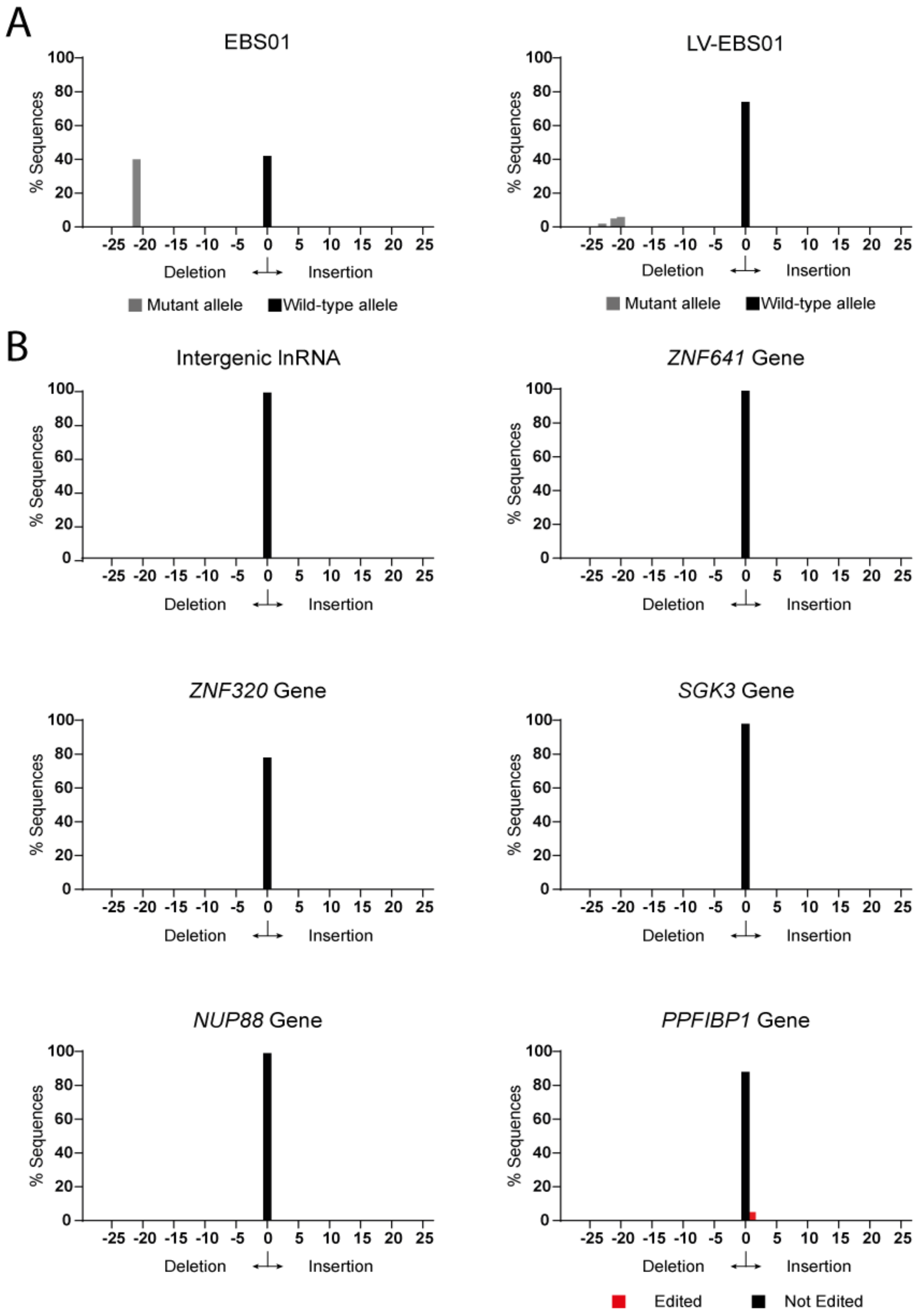

Figure S1: A) Graphic representation of TIDE analysis output after lentiviral vector mediated gene editing. In the not treated sample (EBS01) it is possible to visualize both the wild-type allele (at 0 position) and the mutant allele (with a deletion of 21 nucleotides). In the Lentivirus transduced sample (LV-EBS01) the editing efficiency is 94% with the formation of significant amount of InDels around the cutting site (mutant allele). **B)** Graphic representation of six predicted off-target sites. The continuous expression of the CRISPR/Cas9 components mediated by the lentiviral vector cassettes integration induced an unwanted cleavage in the *PPFIBP1* gene.

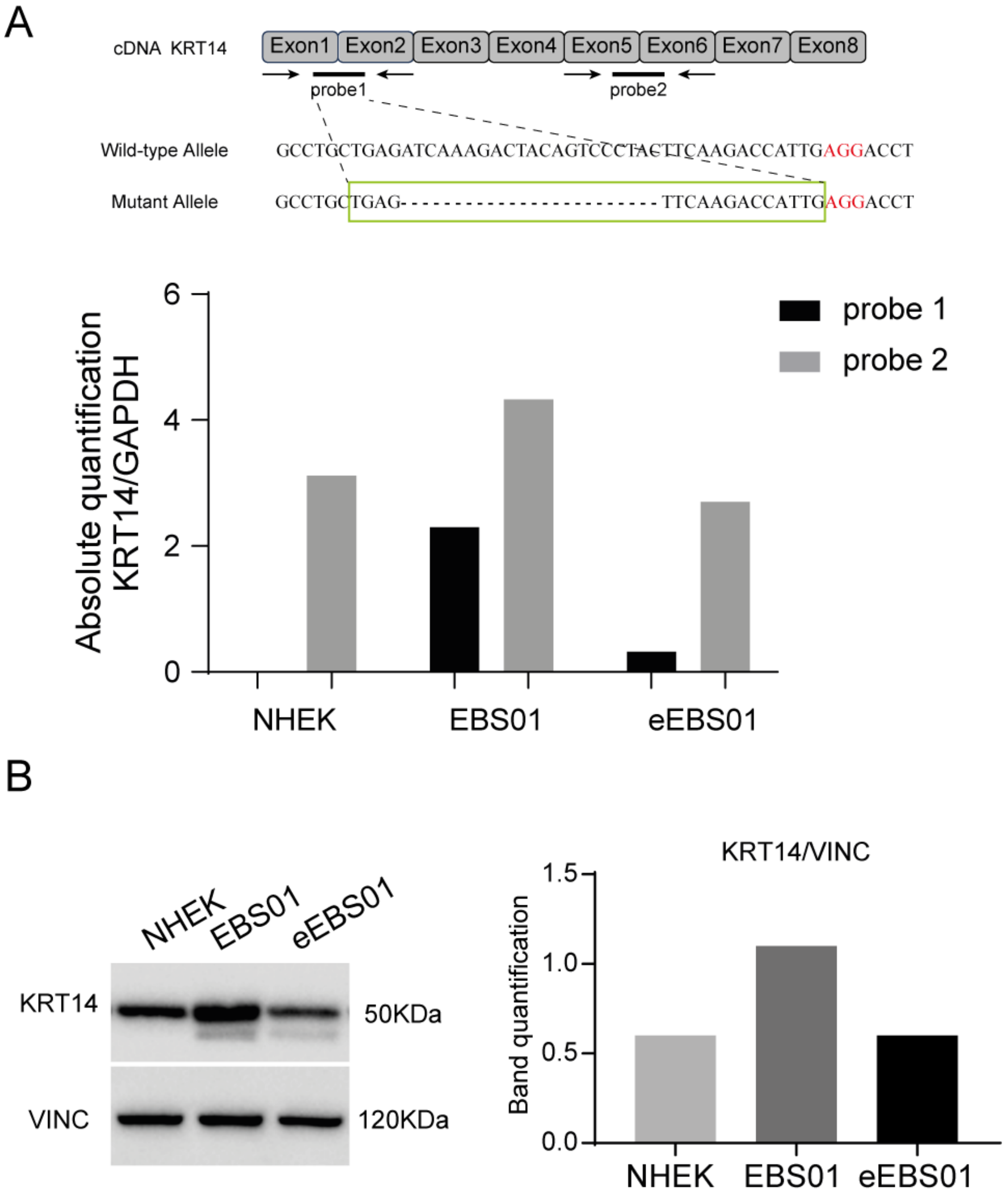

Figure S2: A) Localization of TaqMan probes/primers on the *KRT14* cDNA. Probe 1 (located on exon 1-2) recognizes the mutant allele, while Probe 2 (located on exon 7-8) recognizes both alleles. NHEK expresses only the wild-type *KRT14* allele. EBS01 cells exhibit a higher level of *KRT14* expression compared to NHEK cells when utilizing Probe 2 and express the *KRT14* mutant allele. In eEBS01 cells the expression of *KRT14* using Probe 2 is comparable to that of NHEK cells, whilst the expression of the mutant allele, as detected by Probe 1, is almost fully abolished. **B)** Western blot analysis on NHEK, EBS01 and eEBS01 keratinocytes: higher expression of the K14 protein in EBS01 cells compared to NHEK cells. The K14 expression in eEBS01 cells decreases, as compared to that of control cells (EBS01). Furthermore, the expression level of K14 in the cells after correction returns to the levels observed in NHEK cells.

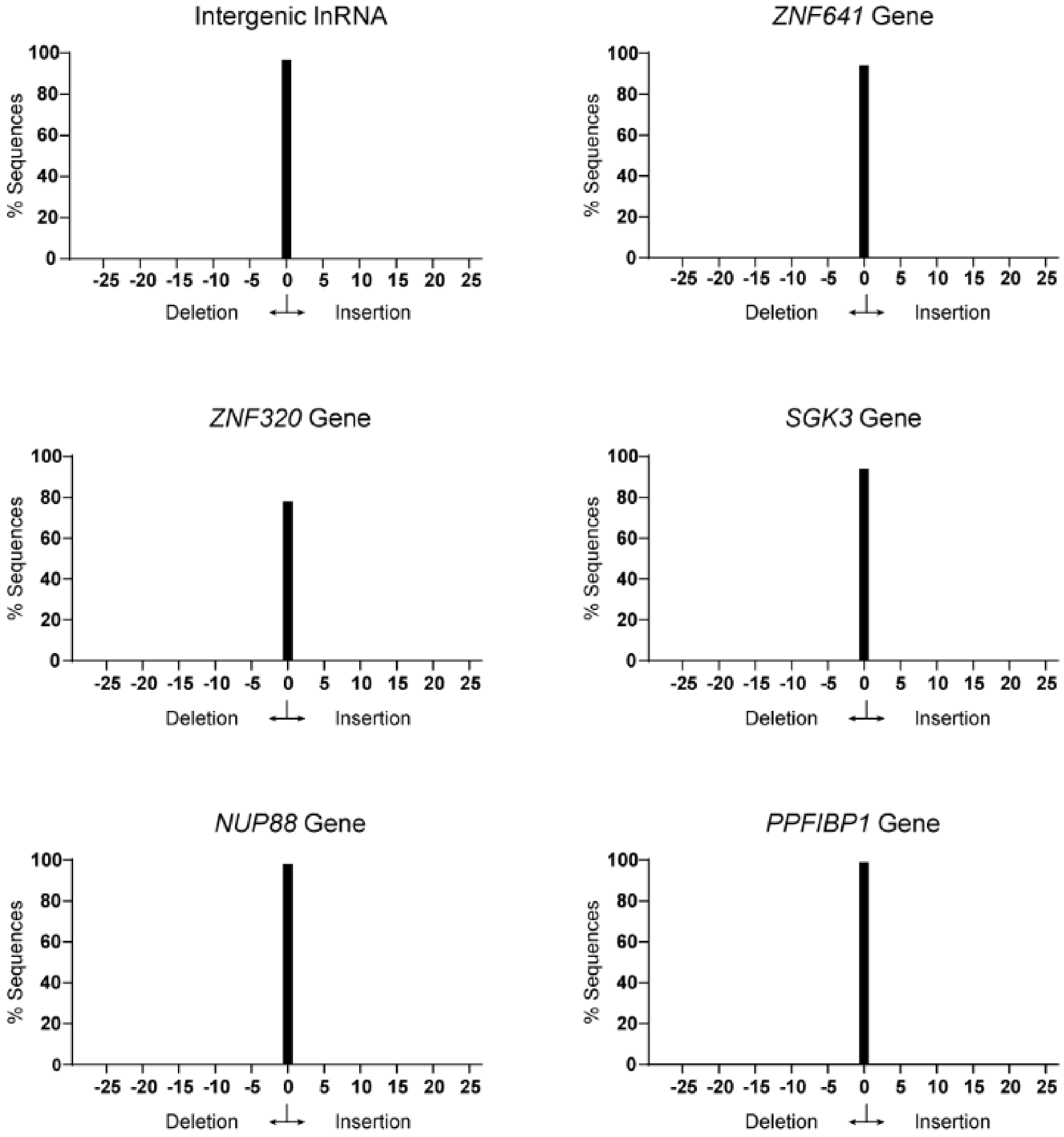

Figure S3: Graphic representation of the selected predicted off-target sites after electroporation mediated gene editing. Due to the transient RNP complex activity, the *PPFIBP1* off-target gene is not detected anymore.

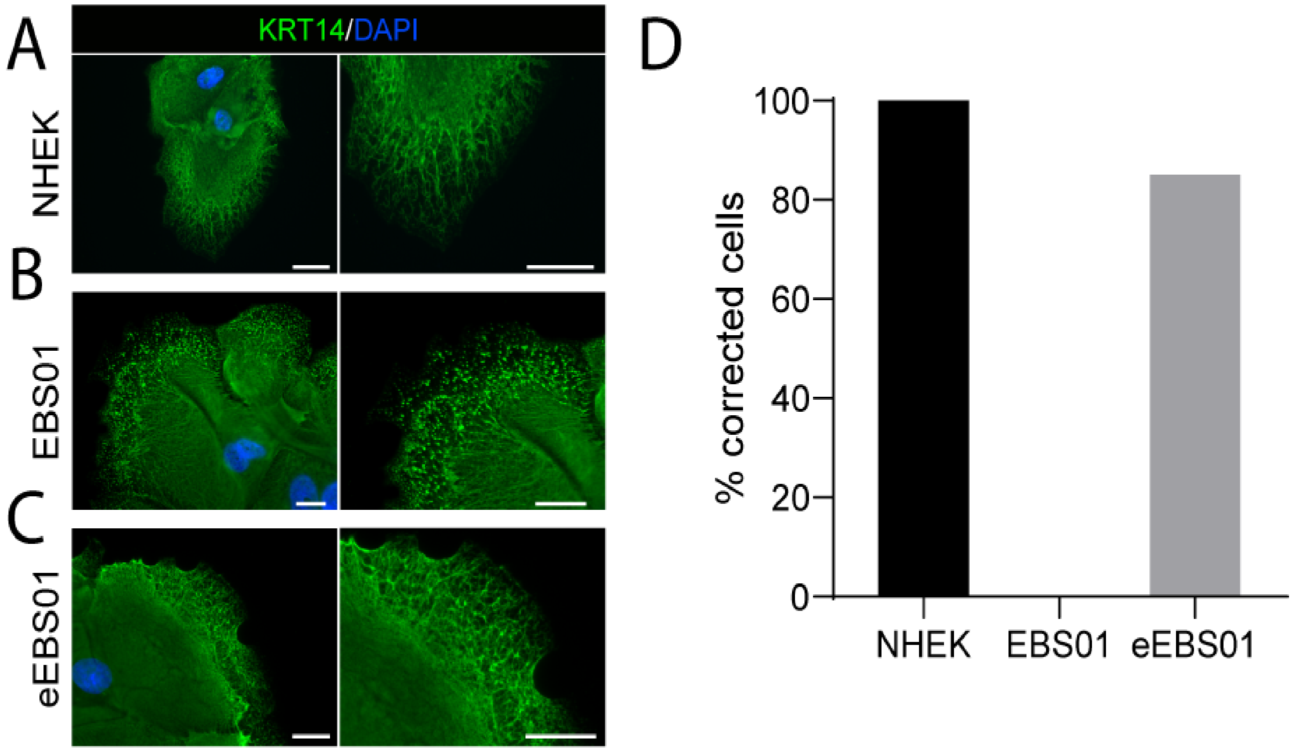

Figure S4: Half coverslip sections on cultured cells were generated with the microscope mosaic function. This assay was performed in duplicates of half coverslips (technical limit of the microscope mosaic function) on NHEK (N=2, counted cells: 636), EBS01 (N=2, counted cells: 722) and eEBS01 (N=4, counted cells 1090) cells. **(A-C)** Representative IF images, scale bar: 20µm. **(A)** NHEK keratinocytes show a correct K14 intermediate filament network. **(B)** EBS01 cells show a fragmented keratin pattern. **(C)** eEBS01 show a keratin pattern indistinguishable from that of NHEK. (**D)** Percentage of cells showing properly assembled intermediate filaments (100% NHEK; 0% EBS01; 86% eEBS01)

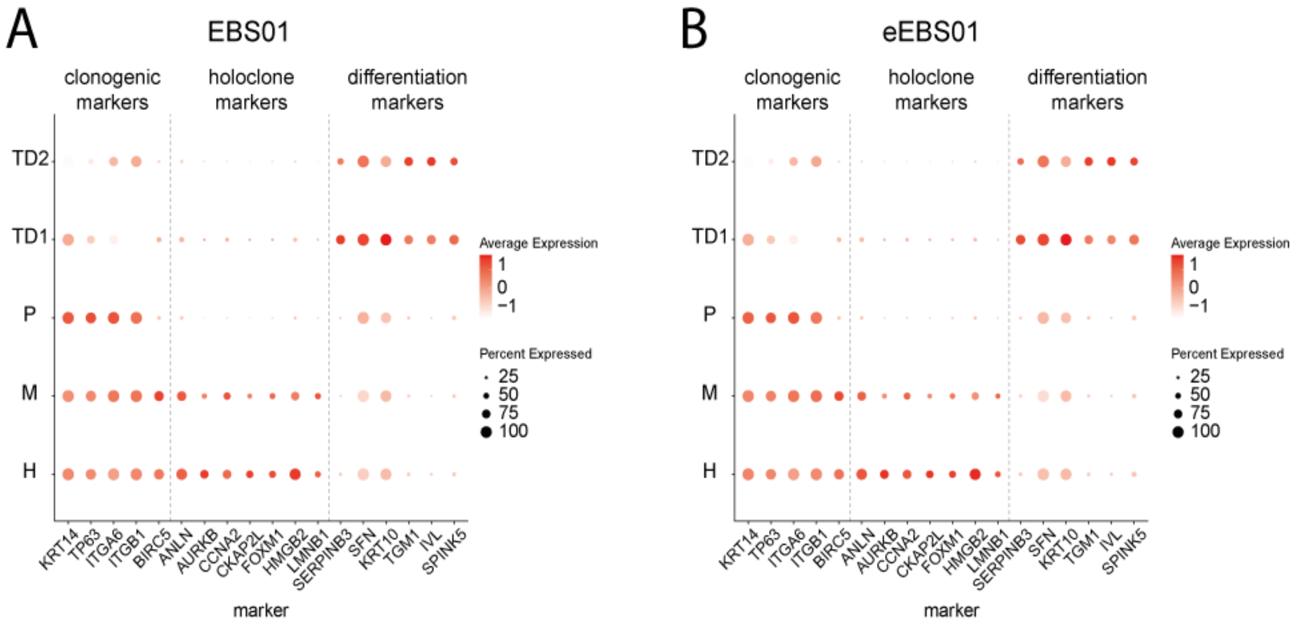

Figure S5: A) DotPlot showing the expression of clonogenic, holoclone and differentiation markers in the five EBS01 keratinocytes clusters. **B)** DotPlot showing the expression of clonogenic, holoclone e and differentiation markers in the five eEBS01 keratinocytes clusters.

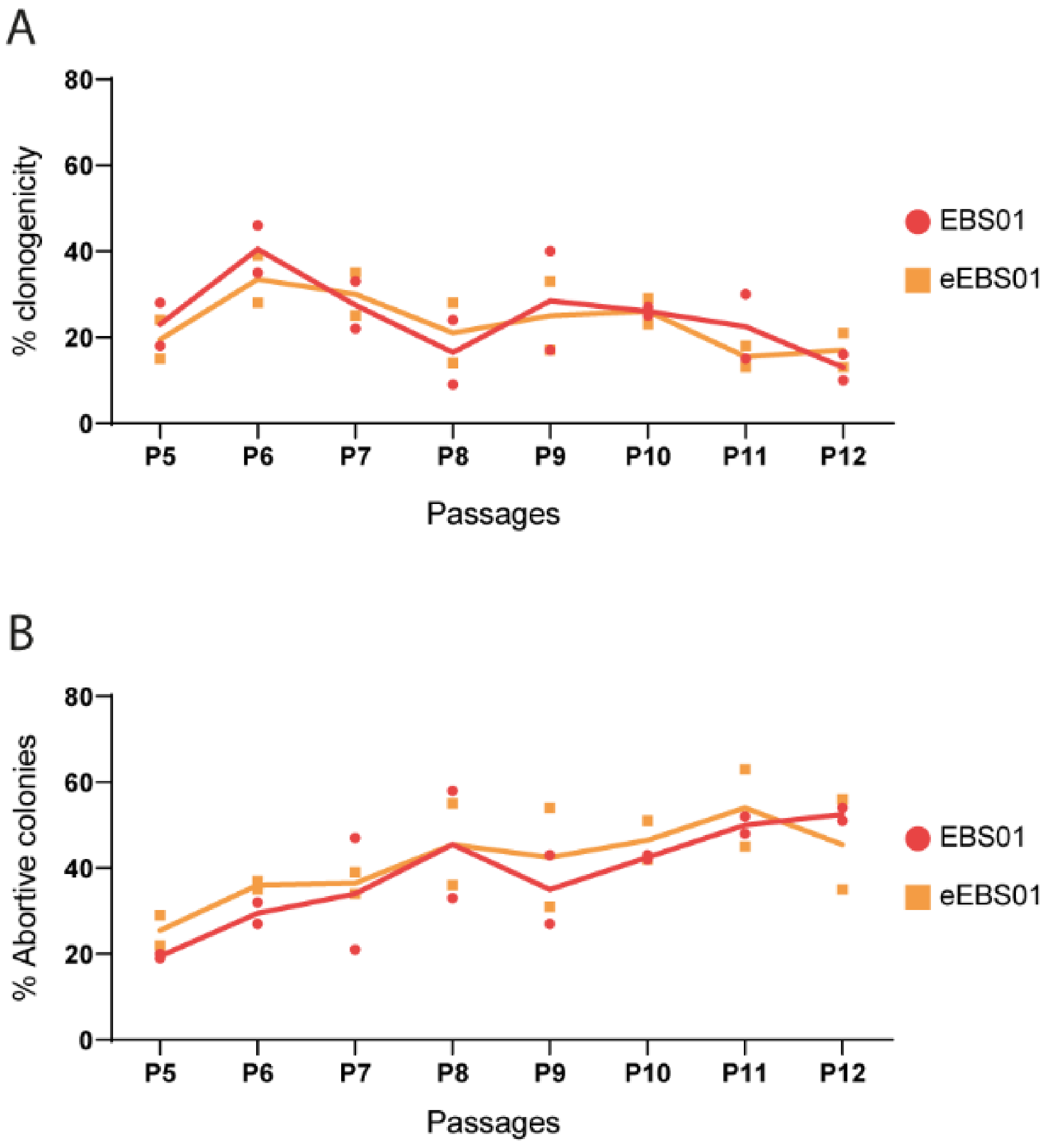

Figure S6: A) Diagram representing the percentage of clonogenic colonies in EBS01 keratinocytes and eEBS01. **B)** Diagram illustrating the percentage of aborted colonies in EBS01 keratinocytes and eEBS01. Both samples show similar trends.

Table S1: PCR Primers and Antibody for IF staining and WB

Name	Type	Sequence 5'-3'
KRT14_seq_del21	For	CTGCTGAGTTCAAGACCATTG
KRT14_seq_wt	For	CAGTCCCTACTTCAAGACCA
KRT14ex1	For	TTGGGGGAGGATATGGTGG
KRT14intr1	Rev	CACTGATCTCACATGGTCTG
KRT14ex1_NGS	For	ACACTCTTTCCCTACACGACGCTCTTCCGATCTTTGGGGGAGGATATGGTGG
KRT14intr1_NGS	Rev	GACTGGAGTTCAGACGTGTGCTCTCCGATCTCACTGATCTCACATGGTTCTG
dsODN_1	dsODN	5Phosp/G*T*TTAATTGAGTTGTCATATGTTAATAACGGT*A*T
dsODN_2	dsODN	5Phosp/A*T*ACCGTTATTAACATATGACAACTCAATTAA*A*C
TrueGuide Modified gRNA_del21		G*C*U*GAGUUCAAGACCAUUG + modified scaffold
Probe GAPDH (Life Technologies)		Cod. 4332649
Probe 1 (KRT14 ex7-8)<br>(Life Technologies)		Cod. Hs00265033_m1
Probe 2 (KRT14ex1-2)<br>Probe-FAM<br>KRT14ex1_F<br>KRT14ex2_R		 TGCTGAGTTCAAGACCATTG<br>GACCTGGAAGTGAAGATCCG<br>CGGGCATTGTCAATCTGCA

Antibody Immunofluorescence	Company	Catalog number	Description	Dilution
Anti-KRT14	BioLegend	905301	Rabbit Polyclonal	1:10000
Donkey anti-Rabbit Ig (H+L) Alexa Fluor 488	Thermofisher Scientific	A21206	Secondary Antibody	1:2000
**Antibody Western blot**	**Company**	**Catalog number**	**Description**	**Dilution**
Anti-KRT14	BioLegend	905301	Rabbit Polyclonal	1:120000
Donkey anti-Rabbit Ig HRP	Santa Cruz Biotechnology	SC-2313	Secondary Antibody	1:5000
Anti-VINC	Sigma-Aldrich	V4505	Mouse Monoclonal	1:5000
Donkey anti-Mouse Ig HRP	Santa Cruz Biotechnology	SC-2314	Secondary Antibody	1:20000

Table S2: Table representation of NGS analysis. Three independent technical replicates post gene editing. Edited and not edited percentages on both wild-type and mutant alleles are calculated on the total number of reads. This data confirmed the allele specificity of this system, with a mutant allele specific gene editing greater than 95% and no editing of the wild-type allele.

	% Wild-type Allele (Not Edited)	% Wild-type Allele (Edited)	% Mutant Allele (Not Edited)	% Mutant Allele (Edited)	% Editing (Mutant Allele)
EXP1	58,4%	0,1%	0,2%	41,3%	99,5%
EXP2	74,5%	0,1%	0,5%	24,9%	98%
EXP3	72,8%	0,1%	1,2%	26%	95,6%

Table S3: GUIDE-seq data

BED Chromosome	Position	BED off-target start	BED off-target end	Total Reads	+Reads	- Reads	+ODN Reads	-ODN Reads	Strand	Off-Target Sequence	Mismatches	GRCh38	% of edited reads predicted via GUDE-seq	% of edited reads detected via NGS analysis
chr9	26986836	26986829	26986851	74365	38960	35405	56717	13509	-	CAGGAGTTCAAGACCATTGTGG	3	Intron IFT74	3,2%	-
chr20	37014292	37014285	37014307	73524	42504	31020	57369	12746	-	CAGGAGTTCAAGACCATTGTGG	3	Intron RBL1	3,1%	-
chr17	64141628	64141612	64141634	71006	16894	54112	58038	9531	+	TGTGAGTTCAAGACCAGTGTGG	3	-	3,0%	-
chr12	118011714	118011698	118011720	59859	24228	35631	48060	8007	+	CAGGAGTTCAAGACCATTGTGG	3	-	2,5%	-
chr7	98337444	98337437	98337459	53372	30917	22455	42549	7497	-	CAGGAGTTCAAGACCATTGTGG	3	Intron BAIAP2L1	2,3%	5,3%
chr14	37288608	37288592	37288614	45483	16999	28484	35440	7597	+	CAGGAGTTCAAGACCATTGTGG	3	Intron MIPOL1	1,9%	4,1%
chr19	52863401	52863385	52863407	43836	19520	24316	34629	6983	+	CAGGAGTTCAAGACCATTGTGG	3	Exon ZNF320	1,8%	6,8%
chr17	41586330	41586323	41586345	42395	0	42395	29967	8660	-	CCCTACTTCAAGACCATTGAGG	4	Exon KRT14	1,7%	Wild-type allele: 0,1%
chr16	62918368	62918351	62918373	24452	14834	9618	20597	2440	+	CAGGAGTTCAAGACCATTGTGG	3	-	1,0%	-

Materials and Methods

Patient, Clinal data and treatments

EBS patient (EBS01) displayed skin lesions to exclusively the acral regions and no mucosal blisters nor erosions were ever reported either by him or his parents. Growth rate and body weight gain were regular according to patient's age. The patient also presented molluscum contagiosum infection near the neck area, then treated in few weeks with daily hydrogen peroxide gel applications. No ocular or dental involvement was detected at baseline evaluation nor was developed at the subsequent follow-up visits.

Notwithstanding, the patient underwent periodic ophthalmological and odontostomatological evaluation according to our integrated care pathway currently in use at the EB Outpatient Clinic of Modena University Hospital. Significant clinical improvement was reported with the topical wound-care regimen (sodium hypoclorite and hydrogen peroxide gel for skin lesions and eosin 2% solution and topical micronizes silver sulfadiazine for exudation).

During follow-up, the family complained about the presence of unremitting blistering of the feet, more evident on the left heel, the dorsal-medial part of the left foot, and the right plantar and peri-malleolar region. A slightly extra-rotated attitude of the hips was noticed, determining relative weight overload on the medial part of the plantar arch. Subsequently the patient also developed flatfoot, bilaterally, treated with specific custom orthotic soles in memory foam and specific daily feet mobilization exercises. to restore the normal anatomy of the plantar vault. At present, the patient is still undergoing regular multidisciplinary follow-up at our center. For genetic counseling, after obtaining informed consent, patient and parents' genomic DNA were extracted from fresh peripheral EDTA-blood samples using the Maxwell 16 LEV Blood DNA Kit on Maxwell 16 System (Promega Corporation, Madison, U.S.A.). The study was approved by the ethics committee of University of Modena and Reggio Emilia and conducted according to the principles of the Declaration of Helsinki (C.E.N. 124/2016). Genetic analysis of the family was performed with a targeted Next-Generation Sequencing (NGS) approach on an Ion Torrent PGM (Thermo Fisher Scientific) using a custom EB AmpliSeq panel including the *ATP2A2, CD151, COL1A1, COL7A1, COL17A1, CSTA, DSP, EXPH5,*

FERMT1, *FREM1*, *GRIP1*, *ITGA2*, *ITGA2B*, *ITGA3*, *ITGA5*, *ITGA6*, *ITGB4*, *ITGB6*, *KRT1*, *KRT2*, *KRT5*, *KRT9*, *KRT10*, *KRT14*, *LAMA3*, *LAMB2*, *LAMB3*, *LAMC1*, *LAMC2*, *MMP1*, *PKP1*, *PLCG2*, *TGM5* genes as well as variant detection, annotation and filtering. Gene variants classified as pathogenic, or of uncertain significance, as well as low coverage regions, were confirmed/sequenced by Sanger sequencing on an ABIPRISM 3130xl Genetic Analyzer (Thermo Fisher Scientific) particularly for *LAMB3* and *ITGB4* genes.

Western blot

Sample destined to western blot analysis were depleted of feeder layer by gentle removal of feeder layer in cold PBS/EDTA (20mM). Keratinocytes colonies were collected after trypsinization. Protein samples from (1 x RIPA buffer, Sigma Aldrich) were supplemented with Protease and Phosphatase Inhibitor Cocktail (Thermo Fisher). Total protein amount was quantified in RIPA extracts using the BCA kit (Pierce). Equivalent quantities of RIPA-solubilized proteins were resolved by SDS-PAGE in 4%–12% NuPAGE Bis-Tris Gels or 10% NuPage Tris-Acetate Gels (ThermoFisher) and transferred 100V at 4°C for 2 hours onto nitrocellulose membrane (Millipore). Membranes were blocked with either 5% (w/v) non-fat milk in PBS supplemented with 0.01% (v/v) Tween-20 (PBS-T) and then probed with the indicated antibodies diluted in blocking buffer (rabbit anti-KRT14 – cod. 905301 from Biolegends and mouse anti-Vinculin clone VIN-.11-5 from Sigma-aldrich (Table S1). Primary antibody-probed blots were visualized with appropriate horseradish peroxidase-coupled secondary antibodies (Santa Cruz). Protein detection was carried out using a chemiluminescent substrate (Clarity Western ECL substrate, BIORAD) and visualized with ChemiDoc. Grey background on the images is homogenously added for graphical purposes.

RNA extraction and droplet digital PCR

Total RNA was isolated from cultured cells using the PureLink RNA Mini Kit (Thermo Fisher). The RNA concentration was quantified using Qubit Fluorometric Quantification (ThermoFisher Scientific). Complementary DNA (cDNA) was synthesized using SuperScript IV VILO (ThermoFisherScientific).

Droplet digital PCR experiments were conducted on the QX200 Droplet Digital PCR System (Bio-Rad). For each ddPCR reaction, the input cDNA was diluted to ensure that 5ng cDNA was input per reaction. ddPCR Supermix for Probes no dUTP and Taqman probes were used following manufacturer instructions (GAPDH cod. 4332649, KRT14 Hs00265033_m1 (probe2), KRT14ex1-ex-2 (probe1, Custom Made) from Life Technologies) (Table S1). Droplets were generated on the QX200 Droplet Generator (Bio-Rad) per manufacturer instructions. Droplet PCR amplification occurred using the following thermocycler conditions, 94°C for 10 seconds; 40 cycles of 94°C for 30 seconds, 60°C for 1 minute; 98°C for 10 minutes; 4°C hold. Amplification was followed by imaging on the QX200 Droplet Reader (Bio-Rad) and analyzed using the QuantaSoft Analysis software package (Bio-Rad).
